# Engineering the Tobacco Etch Virus Protease toward a Platform for Traceless Cleavage Using Distal Site Prediction and Smart Library Design

**DOI:** 10.1021/acssynbio.5c00423

**Published:** 2025-08-23

**Authors:** Martijn P. Bemelmans, Bach-Ngan Wetzel, Florian G. Neusius, Florian Tieves, Christian Schwarz, Ivan Mateljak, Katarzyna Świderek, Vicent Moliner, Miguel Alcalde, Volker Sieber

**Affiliations:** † SynBiofoundry@TUM, 9184 Technical University of Munich, Schulgasse 16, 94315 Straubing, Germany; ‡ NUMAFERM GmbH, Merowingerplatz 1a, 40225 Düsseldorf, Germany; § Evoenzyme S.L., Parque Cientifico de Madrid, 28049, Madrid, Spain; ∥ Biocomp Group, Institute of Advanced Materials (INAM), Universitat Jaume I, 12071 Castello, Spain; ⊥ Institute of Catalysis, 16379 CSIC, 28049 Madrid, Spain; # Chair of Chemistry of Biogenic Resources, Campus Straubing for Biotechnology and Sustainability, 9184 Technical University of Munich, Schulgasse 16, 94315 Straubing, Germany; ∇ Catalysis Research Center, 9184 Technical University of Munich, Ernst-Otto-Fischer-Straße 1, 85748 Garching, Germany

**Keywords:** TEV protease, traceless peptide cleavage, protein engineering, distal site mutagenesis, graph network analysis

## Abstract

Protein tags are vital in biochemical engineering but must be removed from target molecules to prevent compromising effects. Most industrial applications use Tobacco Etch Virus protease (TEVp) for this purpose. However, selectivity at the P1’ position of its recognition site requires N-terminal addition of glycine or serine to noncanonical targets. This residue remains after cleavage, preventing the retrieval of customized peptides with native N-termini. Here, we engineered a TEVp variant (TEVp-C1) with unlocked promiscuity informed by graph network analysis to identify distal positions of influence and smart library design. Compared to state-of-the-art, TEVp-C1 exhibits significantly improved cleavage for 15 of the 20 natural amino acids at P1’ against Switchtag-Teriparatide substrates. Moreover, TEVp-C1 displayed enhanced activity against fluorogenic peptide substrates for five of the most disfavored residues at P1’. Mechanistic analysis revealed that the introduced mutations facilitate the proton transfer step. Altogether, the results highlight the potential of TEVp-C1 as a protease platform for traceless cleavage and demonstrate the feasibility of using tools for the prediction of allosteric interactions to engineer substrate specificities of enzymes via mutations at distal amino acid positions.

## Graphical abstract

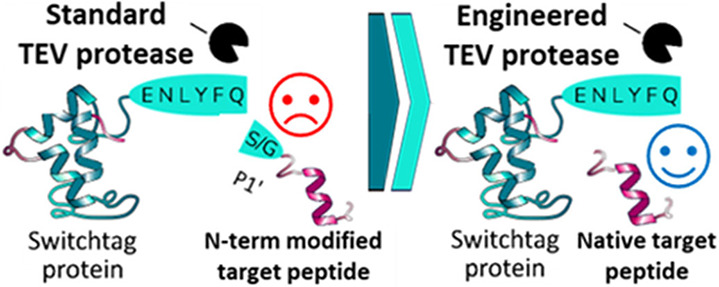

## Introduction

Protein tags have become a powerful tool in protein studies, e.g., as label, for affinity purification or enhanced solubility.^3^ However, it is highly desirable to remove protein tags from target proteins prior to further studies, as they may affect the molecule’s overall structure and biological function.^1^ While this removal can also be achieved by chemical or biochemical approaches, these often require harsh conditions that irreversibly damage the target protein or cause undesired modifications.^2,3^ Moreover, biochemical cleavage strategies are mostly feasible under physiological conditions, but the lack of sequence specificity of proteases, including enterokinase, α-thrombin, or Factor Xa, often induces proteolysis at unintended sites, leading to protein degradation and inactivation.^3,4^ The catalytic domain of the Nuclear Inclusion protein a (Nla) from the tobacco etch virus is a 27 kDa cysteine protease (TEVp) and one of the most widely applied enzymes to remove solubility or affinity tags from fusion proteins.^5^ Typically, protein tags are genetically fused to the N-terminus of target proteins,^6^ and cleavage at the TEVp recognition site in between removes the fusion partner. The canonical recognition sequence of TEVp is ENLYFQ-G/S with cleavage occurring between QG or QS.^7^ The seventh amino acid of the sequence, the P1’ position, is crucial for the substrate specificity of the protease. The key advantage of TEVp in this regard is that it is highly sequence-specific and easy to produce in large quantities in *Escherichia coli* cells, and it is active under a broad range of pH (6.0–9.0) and temperature (18–37 °C) conditions. Altogether, this makes TEVp the enzyme of choice for most industrial applications.^8^

Nevertheless, wild-type TEVp is limited by poor solubility,^9^ slow cleavage rates,^10^ and self-cleavage and autoinactivation^11^ issues. Extensive engineering efforts to optimize TEVp have previously identified the triple mutations T17S, N68D, and I77 V to enhance solubility in directed evolution approaches,^12^ the R203G mutation to increase cleavage rates against substrates with R at the P1’ position,^13^ and the S219N mutation to efficiently prevent self-cleavage near the C-terminus.^14^ Additionally, it was found that C-terminal truncation by deletion of the residues 238–242 yields higher expression in *E. coli* .^15^ Since then, a host of other positions have been investigated for mutation to achieve specific research goals, such as improving catalytic efficiency^10,16^ and tolerance of substrate P1, P2, P3, or P6 positions.^17,18^ A remaining challenge, however, is that P1’ residues other than the canonical G or S are not (well) tolerated by TEVp, leading to a lack or reduction of cleavage activity for noncanonical targets (e.g., E, I, L, V, and P).^1^ While the aforementioned R203G variant improved P1’ tolerance for some targets, most notably R, it still suffers from poor substrate depletion efficiency for others, such as V, and comes at the cost of a significant loss in canonical S or G depletion efficiency. As such, there remains no universally applicable TEVp variant, and the cleavage for noncanonical targets typically still requires the addition of a G or S at the N-terminus of the target protein, which remains after cleavage. While this may not pose a problem for larger proteins, an additional residue can compromise the molecular structure or biological activity for smaller peptides composing 2–50 amino acids.^6^ This is especially true for molecules that exert their function through the N-terminal domain, such as venom peptides.^19^ These drawbacks limit the widespread applicability of TEVp and call for further engineering efforts to enable recognition of any P1’ residue and achieve traceless cleavage of any fusion protein from any target molecule (Figure 1).

**Figure 1:**
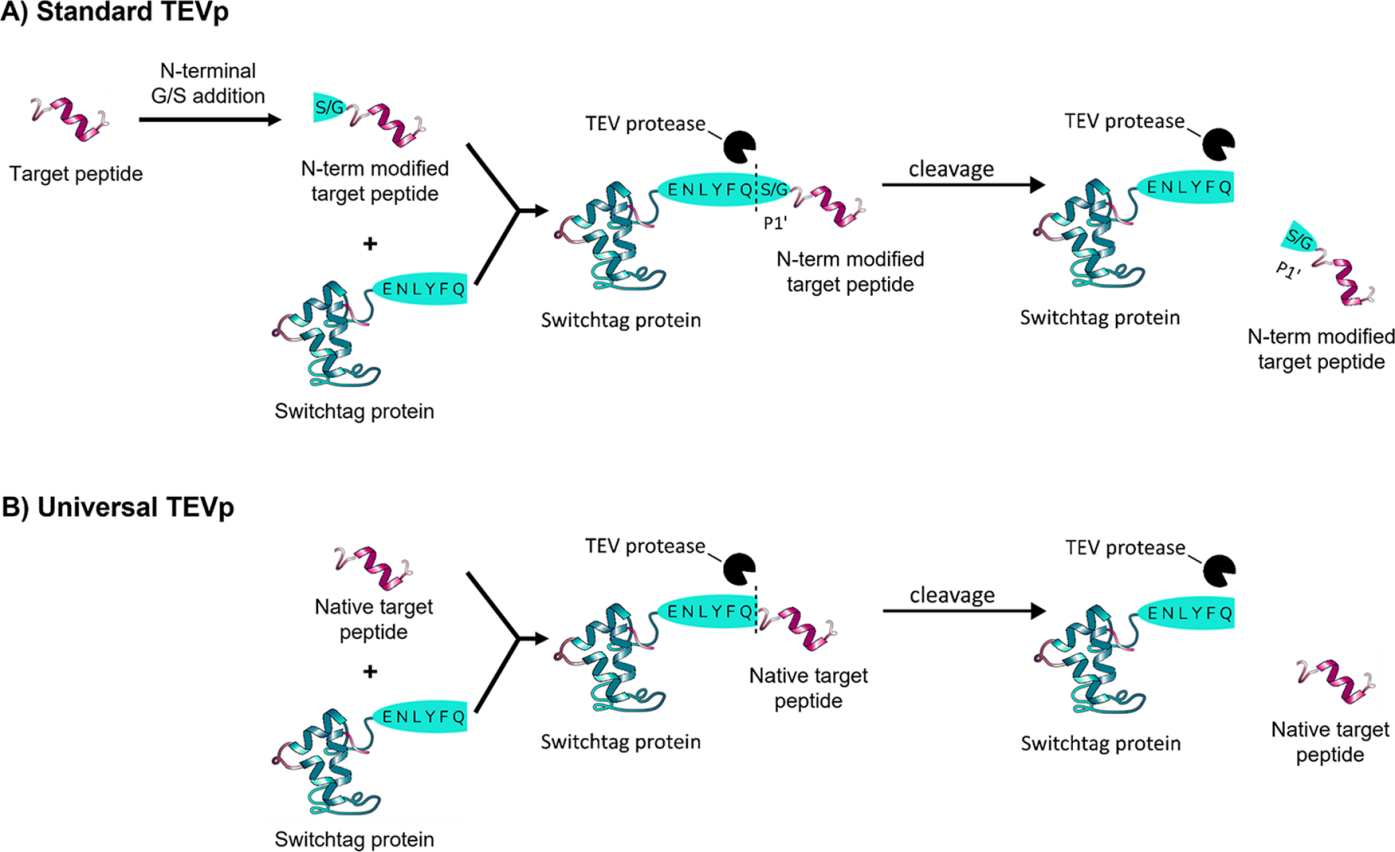
Schematic of the TEV protease cleavage reaction with Numaswitch fusion proteins. (A) Shows the need for N-terminal addition of G or S to target peptides for recognition by TEVp, which remains after cleavage. (B) Illustrates the objective of a “universal” TEVp that can recognize any target peptide N-term, allowing the retrieval of custom target molecules with native N-termini.

In our hands, directly mutating the amino acids of the S1’ site of TEVp has not resulted in variants with a broadened specificity, so we believed it to be important to address positions that are more distant. In this study, we applied SenseNet^20^ in combination with a smart library approach to engineer TEVp to obtain a variant with improved activity toward disfavored amino acids at P1’. SenseNet^20^ is a recently developed *in silico* graph network tool that enables analysis of the evolution of interaction networks between protein residues over time, as sampled with molecular dynamics (MD). Briefly, residues and their interactions are incorporated into nodes and edges, enabling the calculation of node and edge correlation factors (NCF/ECF) to find residues with a strong effect on the conformation of their surroundings. While the main application of SenseNet^20^ so far has been uncovering allosteric residues, we envisioned that its ability to find key influencers of local conformational dynamics could help to identify longer ranging effects in substrate-binding specificities. If so, it could highlight more distal mutant candidates to alter TEVp P1’ tolerance. To broaden the property space covered in the engineering efforts, *in silico* suggestions were used as input for a random library to create a smart library. To progress beyond the state-of-the-art, our starting variant, termed “standard TEVp” (stTEVp), included the main aforementioned engineering efforts. That is, the mutations T17S, N68D, I77 V, R203G, and S219N, and the deletion of the last five C-terminal residues (Δ238–242).

## Results

### Selecting Positions for Mutation

To identify positions of high potential to improve substrate specificity of TEVp toward a mutated P1’ residue, the structural environment of this position was first carefully examined (Figure 2A). Manually mutating P1’S of the canonical recognition sequence in a crystal structure model of a substrate peptide bound to the TEVp (PDB^21^ 1LVB^22^) showed that most mutations leading to poor enzyme activity in experiment^1^ display steric clashes (Supplementary Figure 1). In particular, most clashes occur with side chains of T30, L32, and H46 (pink, Figure 2A). This suggests these residues as clear mutant candidates to improve tolerance at the P1’ position, except for H46, as it is part of the catalytic triad and should thus remain unmodified. The initial analysis furthermore showed that larger amino acids like W and Y clash with the enzyme backbone at the^28^ HTTSLYGIG^36^ β sheet and ^217^FMSKP^221^ loop elements (dark blue, Figure 2A, Supplementary Figure 1). Since these backbone clashes cannot be resolved by mutating the amino acids in these elements, more cryptic mutant candidates at more distal positions were sought.

**Figure 2:**
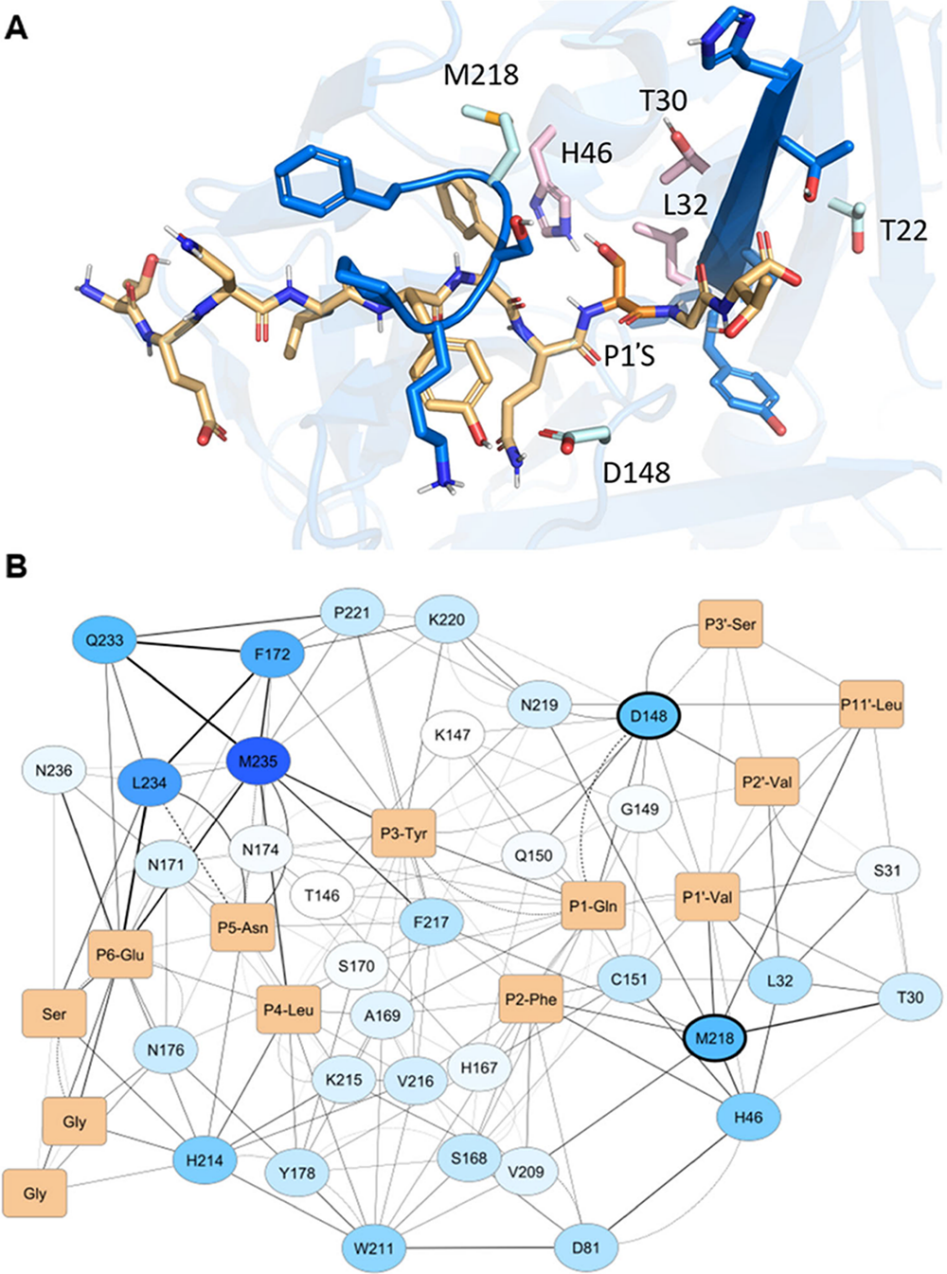
Structural and SenseNet analysis of TEVp. (A) Depicts the binding site of TEVp (blue) with an example substrate (orange, PDB^21^ ID 1LVB^22^), highlighting the peptide P1’S (dark orange). TEVp residues T30, L32, H46 (pink),^28^ HTTSLYGIG^36^ β sheet and ^217^FMSKP^221^ loop (dark blue), which often sterically clash with P1’ during *in silico* mutagenesis of the Serine, are also highlighted. SenseNet^20^ and further analysis identified T22, D148, and M218 (light cyan) as mutant candidates to improve P1’ tolerance. (B) Shows the interaction network between stTEVp (ovals) and a Switchtag-Teriparatide substrate (orange rectangles) with P1’V obtained from SenseNet^20^ analysis. Protease residues are colored from white to dark blue as their node correlation factor increases, and edges are depicted from thin to thick as their edge correlation factor increases. Solid lines indicate hydrophobic contacts, while dashed lines show H-bonds. D148 and M218 (encircled in bold) show up as key residues impacting tolerance of P1’V.

For this stTEVp was subjected to a graph network analysis with SenseNet.^20^ Herein, stTEVp was first simulated using MD with a Switchtag-Teriparatide fusion protein substrate (see Methods section) bearing a P1’V instead of the canonical P1’S. This mutant was chosen as an initial case study since V is relatively small and should thus not require big conformational changes to be tolerated, yet it was identified as one of the disfavored residues at the P1’ position by previous studies.^1,13^ All residues were represented as nodes and the interactions between them as edges in CytoScape (v3.8.2).^23^ SenseNet^20^ was then used to calculate the edge and node correlation factors (ECF/NCF), which enabled visualization of residues that strongly affect the conformation of their surroundings (Figure 2B). Focusing on the peptide sequence and its nearest neighbors, D148 and M218 were identified as high-potential positions to enhance P1’V tolerance. These residues have a relatively strong conformational impact on their surroundings (darker blue nodes, Figure 2B) and make several interactions with a strong conformational effect (thick lines, Figure 2B). Moreover, they are in direct contact with the P1’V (P1’-Val, Figure 2B). M218 additionally moderately affects T30 and H46’s conformations, which were expected to cause clashes for some P1’ mutations based on the initial mutation analysis (Supplementary Figure 1).

To further assess the likelihood of these positions impacting the tolerance of P1’V, the structural relevance of D148 and M218 in the TEVp was next assessed visually (Figure 2A). This showed that D148 and M218 are indeed located close to the P1’ position, being roughly below and above it, respectively. Moreover, M218 is part of the ^217^FMSKP^221^ loop that was identified before as an important element in P1’ tolerance. Finally, in conjunction with the network information, T22 was identified as an interesting mutation candidate to affect P1’ acceptance. This residue is located directly behind T30, which was already selected as a mutation candidate and may therefore have synergistic effects if mutated along with it.

### Mutagenesis Strategy and Smart Library Design

With positions 22, 30, 148, and 218 selected for mutation, the degree of randomization had to be set next. Simultaneous full saturation mutagenesis with allowance of every possible amino acid at these positions would have resulted in a library of 160,000 variants, which was far from feasible to analyze. Instead, the screening effort was sought to be in the range of a few hundred variants. Accordingly, the number of mutation candidates was limited to four or five per position, and unreasonable exchanges were rationally excluded to obtain a “smart” library, limiting the variability to an extent that it contains a high proportion of desired variants. The choice of amino acids to be included at each position was made based on the potential effects of their side chain structures on the surroundings and in order to sample diverse properties. As the main clashing contact for P1’V, T30 was first selected for mutation to the smaller serine and alanine to reduce steric hindrance. The introduction of a negative charge with aspartate or a larger hydrophobic group with isoleucine was also selected, as this was expected to move the P1’ residue to the side by additional interactions with surrounding parts of the protein. Since T22 has no direct interaction with the P1’ position but instead interacts with the clashing T30, it was selected for mutation to the slightly smaller serine. This was posited to maintain H-bonding ability yet create space for T30 to move closer and enlarge the binding site to tolerate bigger residues at the P1’ position. The bulkier and aromatic tyrosine was additionally chosen for position 22, as it also maintains H-bonding ability and was anticipated to move T30 to the side due to its larger size, again expanding the binding site. Likewise, the more hydrophobic valine and isoleucine (the former being isoelectronic to threonine, the latter slightly larger) were selected to be tested for their effect of pushing T30 aside or allowing an improved interaction with a possible alanine at position 30. For D148, the whole range of possible interactions was considered with either a positive (arginine) or negative (aspartate, wild type) charge, removing the charge but keeping the H-bonding ability (asparagine) and introducing a hydrophobic (isoleucine) or aromatic (tyrosine) residue. As alternative amino acids for M218, bulkier or aromatic residues were selected (leucine, isoleucine, and phenylalanine) since the interactions of this position are predominantly hydrophobic. In addition, lysine was permitted, as it also has substantial hydrophobic properties with its four methylene groups and the option of forming a salt bridge with a potential aspartate at position 30. Altogether, this selection of amino acids considered per position (Figure 3A) led to 320 possible residue combinations. Based on this, the library was prepared by GeneSoeing^24^ using a mixture of specific and degenerated primers. The quality of the library was then confirmed by sequencing 20 clones, which all showed different combinations of residues at the relevant positions.

**Figure 3:**
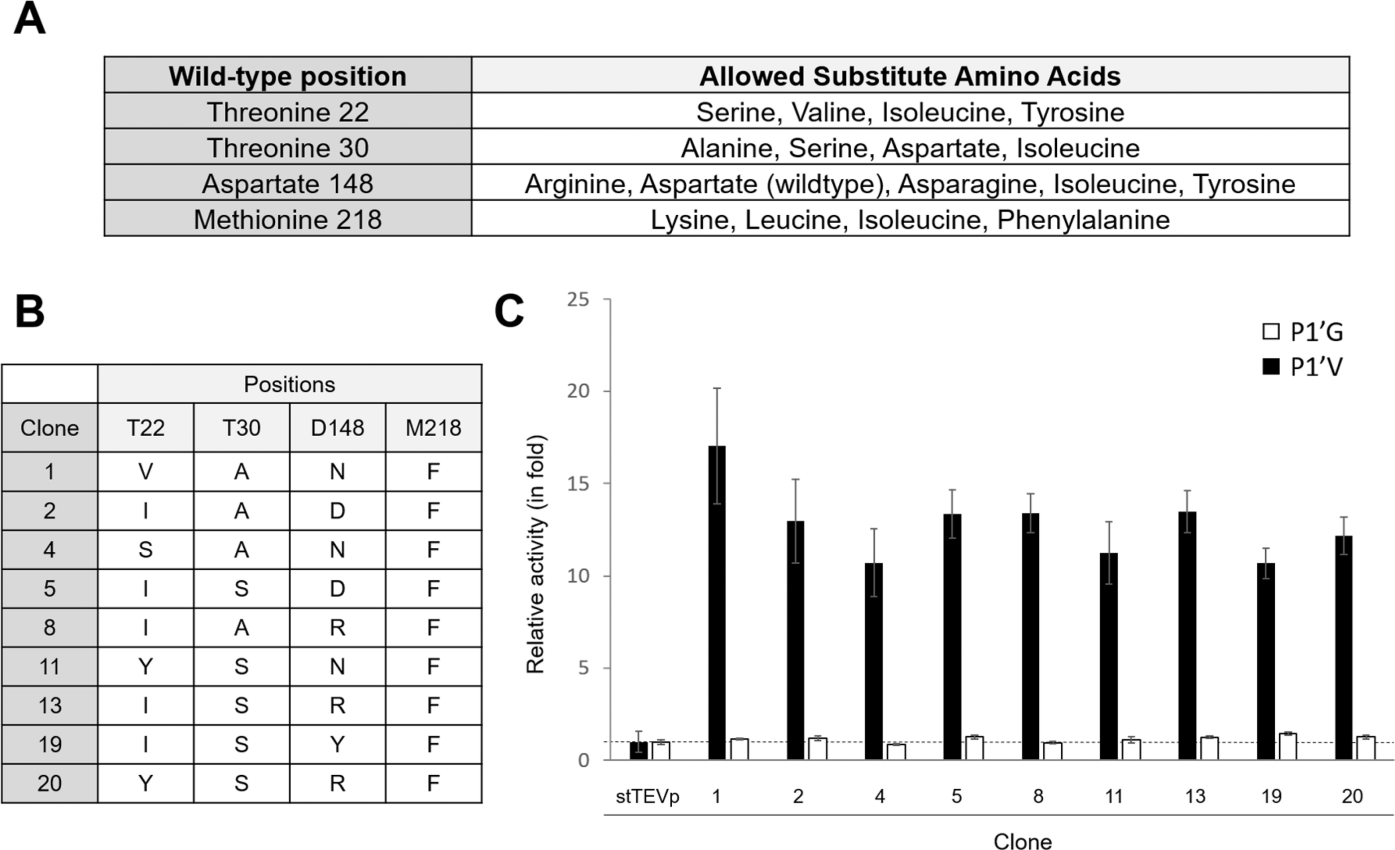
Initial TEVp clone screening against fluorogenic peptides to assess cleavage efficiency against P1’V. (A) Shows the mutations allowed per chosen position, while (B) lists the observed mutations per clone in the smart library, and (C) displays the relative cleavage activity of stTEVp and the tested clones against P1’G (white) and P1’V (black). Clone 1 shows the highest relative improvement for P1’V.

### Screening Performance on the Smart Library

To test the effect of the selected mutations on the TEVp cleavage efficiency, 896 stTEVp clones from the smart library were analyzed. With 320 possible variants (i.e., residue combinations), this 2.8 fold oversampling of clones results in a library coverage of 94%. All clones were subjected to fluorogenic peptide substrates containing the sequence ENLYFQXGGK labeled with an EDANS fluorophore and Dabcyl quencher at both ends upon which release of EDANS was measured to quantify cleavage. For the initial P1’V test case, the ENLYFQ-G peptide (control screening) and ENLYFQ-V peptide (target screening) were used. In short, library variants containing cells were grown in 96-well microplates, after induction, and protein expression cells were lysed. The cell lysate was transferred to a solution containing the peptide substrate, and the development of fluorescence was analyzed immediately. In total, 896 TEVp clones were screened with both fluorogenic peptides, including 58 clones of parental stTEVp, which achieved an acceptable coefficient of variation (CV) of 15% (Supplementary Figure 2). A remarkable number of 119 mutant clones (13%) showed higher activity than the CV threshold (21%), of which the 20 best performing clones for the ENLYFQ-V peptide were selected for a rescreening process (Figure 3B,C). Out of these, nine unique mutants showed significantly higher cleavage activity against the ENLYFQ-V peptide compared to that of stTEVp, while activity for the ENLYFQ-G peptide was preserved in all clones (Figure 3C). The other 11 clones were repetitions, which was not surprising considering the oversampling of the library and which demonstrates the high quality of the screen. Looking at the mutations present in the nine screening hits (Figure 3B), position 218 notably always contained phenylalanine, showing the importance of this residue. At position 30, only alanine and serine were present, demonstrating the need of making this residue smaller. The other two positions were more flexible and allowed almost all exchanges. Among mutant winners, especially clone 1 (TEVp-C1) stood out, with a 17-fold improvement for the ENLYFQ-V peptide compared to stTEVp (Figure 3C).

### Validation of the Screening Hits

To verify the observed enhanced cleavage efficiency of the best-performing clone (TEVp-C1) for P1’V in the library mutant screenings and study the unlocked tolerance of this variant for other unfavorable amino acids at the P1’ position, rescreening was performed with purified TEV proteases (>95% purity, Supplementary Figures 3 and 4). stTEVp and TEVp-C1 variants were tested against control substrate ENLYFQ-G and seven ENLYFQ-X peptides harboring the disfavored K, R, E, T, V, I, or L at P1’. The specific activities of TEVp-C1 toward 25 μM peptide substrates were compared to stTEVp (Figure 4A). While stTEVp showed little to no activity against peptides with R, E, V, I, and L at P1’, TEVp-C1 displayed markedly higher activities against these substrates, indicating improved tolerance for charged and hydrophobic amino acids. Comparable activities for P1’G, K, and T substrates were achieved for both stTEVp and TEVp-C1 variants.

**Figure 4:**
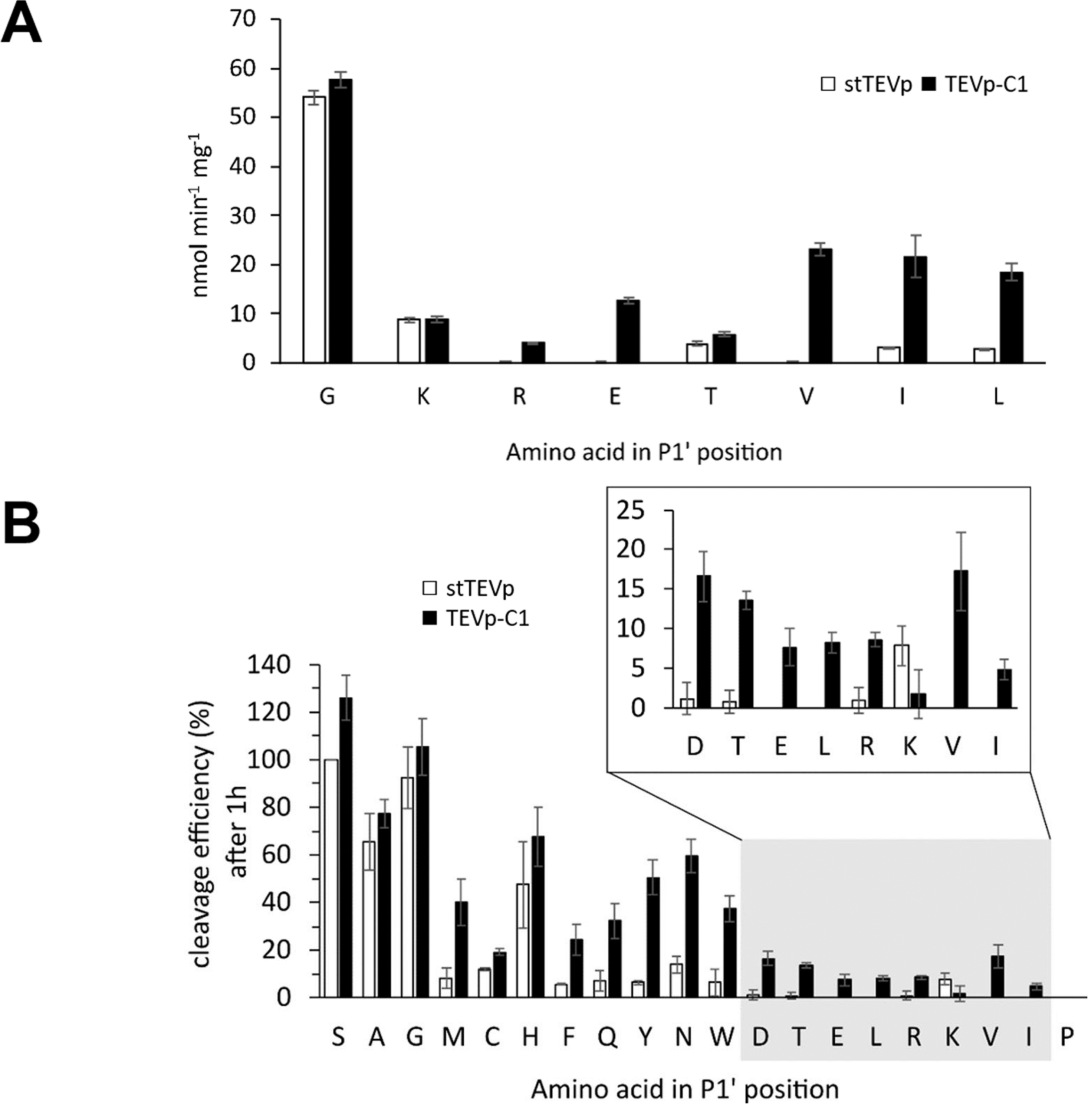
Screening of stTEVp and TEVp-C1 cleavage activity against fluorogenic peptides and Switchtag-Teriparatide substrates. (A) Shows the specific activities of stTEVp and TEVp-C1 against 25 μM fluorogenic peptide substrates with different amino acids at the P1’ position. (B) Displays the release of Teriparatide variants from Switchtag after proteolytic cleavage with stTEVp and TEVp-C1 for different P1’ residues, quantified by chromatographic elution peak integration (OpenLab ChemStation data software, Agilent) after RP-HPLC. Shown are the relative cleavage efficiencies (in %) compared to stTEVp with ENLYFQ-S (set to 100%). Error bars indicate the standard deviation; measurements were performed in triplicate (A, B).

Encouraged by the broadly enhanced cleavage activities for disfavored substrates, a follow-up screen was performed to investigate the tolerance of TEVp-C1 against all 20 canonical amino acids. N-terminal Switchtag fusion proteins were employed for this screen, which are composed of a Switchtag protein and a Teriparatide peptide with a unique N-terminal residue connected through a TEVp recognition site (Figure 1). Cleavage efficiency was then determined for all 20 substrates by analyzing the amounts of Teriparatide peptide released from the Switchtag protein by stTEVp or TEVp-C1 using RP-HPLC (Figure 4B). The identities of released Teriparatide targets were confirmed by mass spectrometric analysis (Supplementary Figure 5). Considering the size of these structures, the recognition site is assumed to be less accessible compared to the short fluorogenic peptides used in previous screening, rendering them more suitable substrates to assess cleavage efficiencies under future application conditions. For 15 substrates (P1’ S, M, C, F, Q, Y, N, W, D, T, E, L, R, V, and I), the cleavage efficiency of TEVp-C1 after 1 h was significantly higher compared to stTEVp (Figure 4B).

The biggest relative improvements in cleavage activity were observed for substrates bearing an E, L, V, or I at the P1’ position, which did not register any cleavage for stTEVp after 1 h but were tolerated by TEVp-C1. Regarding the other substrates, cleavage efficiency increased the most for M, Y, N, and W at the P1’ position when comparing TEVp-C1 to stTEVp cleavage (4–8 fold). The lowest cleavage efficiencies of TEVp-C1 were registered for substrates carrying P1’K (similar activity as stTEVp, *p* = 0.07, two-tailed *t* test) and P1’P. These data align well with the results from the microtiter plate assay, where superior performance of TEVp-C1 against fluorogenic peptides with P1’ E, V, I, and L was observed (Figure 4A). To study the long-term cleavage efficiencies, RP-HPLC analysis was also performed after 16 h of incubation (Supplementary Figure 6). This highlighted even further the superior cleavage activity of TEVp-C1 against the majority of disfavored P1’ amino acids (e.g., F, Q, Y, N, D, T, V, I). In particular, for P1’ W, the cleavage efficiency was drastically improved for TEVp-C1 (90% compared to 9% for stTEVp).

Separately, cleavage efficiency was studied by SDS-PAGE after 1 and 16 h of incubation (Supplementary Figure 7) and densitometric quantification of fusion protein and Switchtag protein bands with the ImageJ^25^ software.^26^ This confirmed the RP-HPLC results, as similar cleavage efficiencies were observed. Only for P1’ C contradictory results were observed, as higher cleavage rates were obtained compared to RP-HPLC analysis both after 1 h (stTEVp: 33% vs 12%, TEVp-C1:48% vs 19%, Figure 4B, Supplementary Figure 7B) and 16 h of incubation (stTEVp: 77% vs 17.5%, TEVp-C1:85% vs 23%, Supplementary Figure 6, 7C). Further studies showed that proper product recovery was hampered by the formation of cysteine dimerizations during the cleavage reaction. The addition of the reducing agent DTT in the cleavage reactions inhibited disulfide bridge formations and considerably increased the product recovery in RP-HPLC analysis.

### Understanding the Improved Tolerance of TEVp-C1

To uncover why TEVp-C1 unlocked promiscuity at the P1’ position compared to stTEVp, long MD simulations were performed under the experimental conditions of this study with classical force fields at the noncovalent protein:substrate reactant complex (see Methods section). The stTEVp and TEVp-C1 variants were simulated in the presence of the fluorogenic peptides used in the experiment, containing P1’G or P1’V, resulting in four simulations of 6 μs each. Following the general molecular mechanism of peptide hydrolysis catalyzed by cysteine proteases,^27^ both substrates adopted a proper pose in either TEVp variant for nucleophilic attack of the cysteine to the carbonyl carbon of the peptide (average distance Sγ^C151^···C^scissile‑bond^ = 3.5 Å, Figure 5A,B).

**Figure 5:**
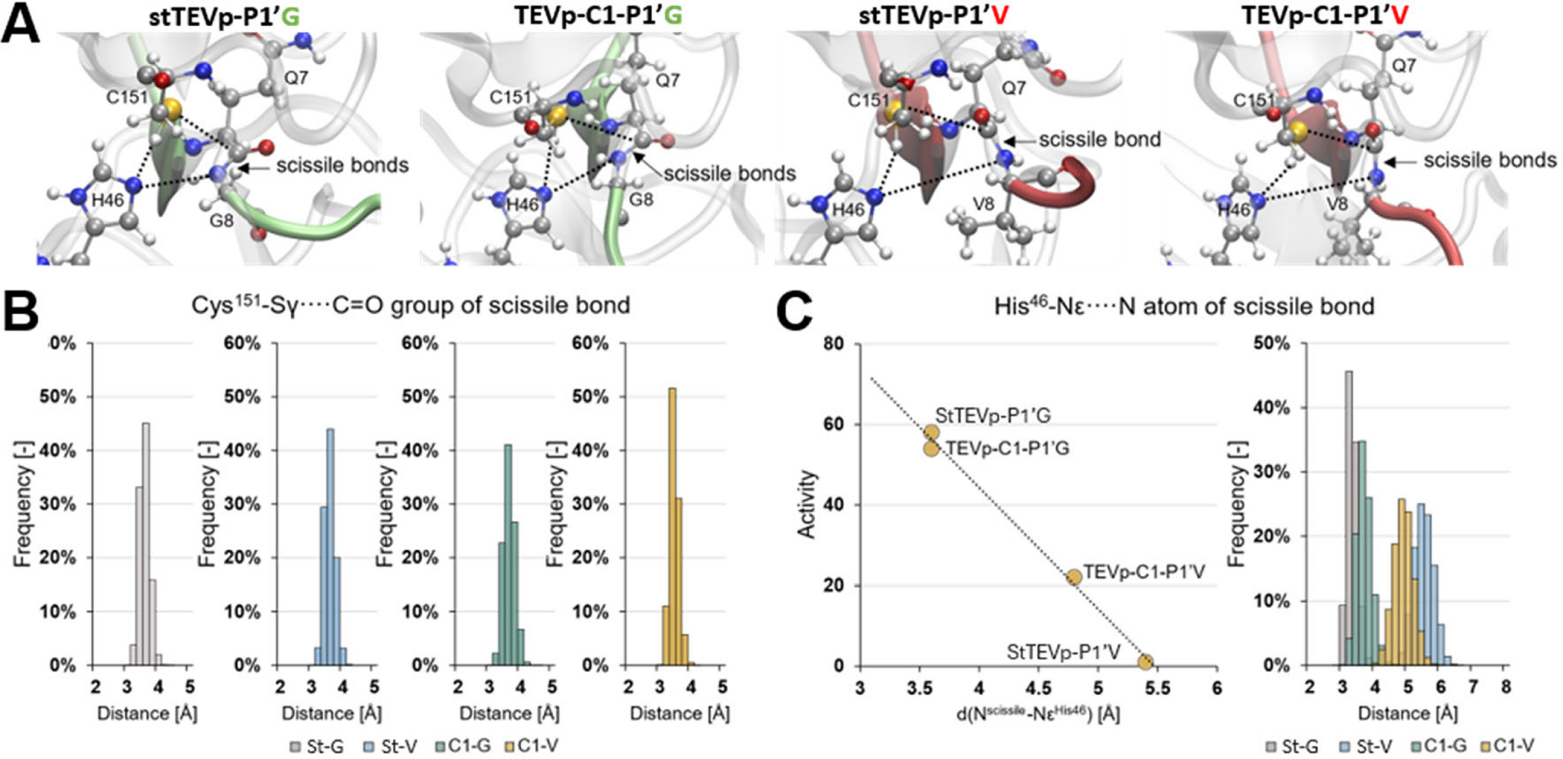
TEV protease activity vs enzyme:peptide binding. (A) Shows representative structures of the active site in the stTEVp and TEVp-C1 systems. Key interatomic distances involved in the proteolysis reaction are indicated as dashed lines. (B) Displays a population analysis of the Nε···N^scissile‑bond^ distance in the active site of stTEVp and TEVp-C1 variants with glycine and valine at the P1’ position (St-G, St-V, C1-G, and C1–V). (C) Shows a linear correlation between the Nε···N^scissile‑bond^ distances and the protease activities as well as a sum of the population analyses of the four systems.

However, the subsequent proton transfer step from His46 to the nitrogen atom of the scissile peptide bond of the substrate is affected differently in the studied systems. Substituting P1’G to the bulkier P1’V increased the average distance between the Nε^His46^ and the N^scissile‑bond^ atoms in stTEVp from 3.5 to 5.5 Å, while only rising from 3.6 to 4.8 Å in TEVp-C1 (Figure 5C). Plotting the experimentally measured enzymatic activity against this distance shows that the cleavage efficiency linearly decreases as Nε^His46^···N^scissile‑bond^ grows (Figure 5C). Accordingly, cleavage activity is highest in stTEVp-P1’G and TEVp–C1-P1’G (distance 3.5–3.6 Å), moderate in TEVp–C1-P1’V (distance 4.8 Å), and absent in stTEVp-P1’V, where the distance of 5.5 Å is too large to allow direct proton transfer.

Analysis of the interaction network between the protein and substrate shows that the mutations introduced at positions 22, 30, 148, and 218 did not affect the overall binding pose of the substrate aside from the C-termini (Figure 6A). However, a significant influence on the protein residues surrounding the substrate P1’ position (i.e., the S1’ cavity) was found, highlighting the impact of the D148N mutation in particular (Figure 6B). In stTEVp, the combination of electrostatic and van der Waals interactions between D148 and the substrate is largely unfavorable. This is greatly improved, and even a favorable interaction is established in TEVp-C1 between N148 and the substrate. For substrates with a P1’G, the interaction with residue 148 is favorable for D or N, though D148 is preferred.

**Figure 6:**
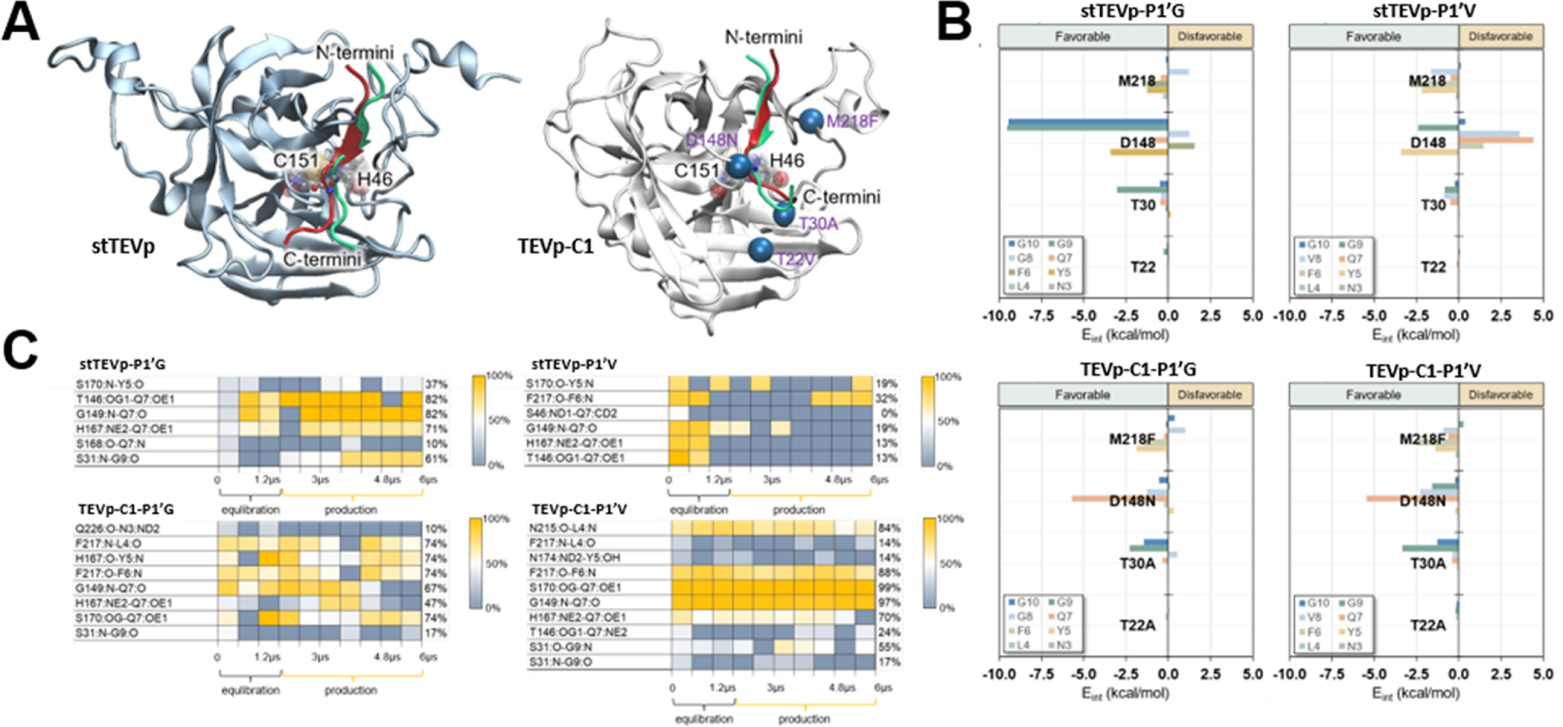
TEV protease:peptide interactions. (A) Depicts a superposition of the P1’G and P1’V peptide substrates in stTEVp and TEVp-C1 variants. The 4 mutations on TEVp-C1 are shown as blue spheres. (B) Shows interactions energy (in kcal/mol) computed between substrates and the residues that were chosen for mutation in the TEVp-C1 variant. (C) shows H-bond contacts established between core residues of the P1’G and P1’V substrates in both stTEVp and TEVp-C1 observed during MD simulations.

Regarding the H-bonding network (Figure 6C), the most preserved interactions in stTEVp-P1’G are those formed with the substrate P1 position (T146:Oγ-Q7:Oε, 82%, backbone G149:N-Q7:O, 82%, and H167:Nε-Q7:Oε, 71%) and the backbone of substrate P2’G (S31:N-G9:O, 61%). All of these key interactions are lost when P1’G is substituted to P1’V, though a new relevant contact is established with the backbone of the substrate P2 position (F217:O–F6:N, 32%).

Comparing TEVp–C1-P1’G to stTEVp-P1’G, the H-bonding pattern is altered by the mutations but remains substantial. Despite losing the T146:Oγ-Q7:Oε bond, the protein still forms key interactions with P1Q (S170:Oγ-Q7:Oε, 74%, backbone G149:N-Q7:O, 67%, and H167:Nε-Q7:Oε, 47%) and the backbone of P2’G (S31:N-G9:O, 17%). In addition, new H-bond contacts are established with the backbone of the substrate at positions P4 (F217:N-L4:O, 74%), P3 (H167:O–Y5:N, 74%), and P2 (F217:N–F6:N, 74%) to secure the substrate in place. For TEVp-C1, substitution of P1’G to P1’V no longer results in loss of the key interactions with P1Q and P2’G. Indeed, it retains and even strengthens its bonds with P1Q (S170:Oγ-Q7:Oε, 99%, backbone G149:N-Q7:O, 97%, H167:Nε-Q7:Oε, 70%, and T146:Oγ-Q7:Oε, 24%) and P2’G (backbone S31:O-G9:N, 55%, and S31:N-G9:O, 17%). Furthermore, the backbone H-bond between F217:N–F6:N (88%) observed in stTEVp-P1’V and a novel H-bond between N215:O-L4 (84%) are established for TEVp-C1.

## Discussion

Here, we investigated how to improve tolerance at the P1’ position of TEVp to circumvent the need for N-terminal addition of glycine or serine to the target peptide that remains after cleavage for noncanonical targets. The SenseNet-informed structural analysis highlighted four partially distal positions for high-impact mutations, which were explored in a smart library to yield TEVp-C1. We observed that this final variant displayed elevated cleavage rates not only for disfavored residues at P1’ but even for most of the 20 natural amino acids. Given the unlocked promiscuity of TEVp-C1 found in different tag protein-target substrate constructs, this improved TEVp variant shows promise for use as a generalist protease to produce peptides and proteins with native N-termini.

To the best of our knowledge, a TEVp variant with collective mutations T22 V, T30A, D148N, and M218F has not been explored before. Nevertheless, some of the positions explored for mutagenesis in this study were previously found to affect TEVp cleavage. For instance, Yi et al.,^18^ observed that including a mutation at position 148 (along with 120, 173, 177, 218, and 219) altered the acceptance of different residues at the substrate P1 position. Aiming to improve tolerance at multiple positions of the canonical recognition site, Packer et al.,^17^ similarly found that including mutations at positions 148 and 218 (along with 171, 176, 178, 209, and 211, among others) respectively improved tolerance at the P1 and P2 substrate positions. Interestingly, in later evolution stages of their variants containing a mutation at position 218, T30A emerged in epistasis. This concurs with our SenseNet^20^ analysis revealing that positions 30 and 218 are influenced by each other, and the evolution observed in our smart library, where only a smaller serine or alanine was allowed at position 30 upon M218F. Considering that D148 and M218 are in the direct vicinity of the P1 and P2 substrate residues, it coheres that these positions were explored for mutation in other studies to improve tolerance for TEVp at these positions. However, here we show that these positions also strongly impact the tolerance at the P1’ position. Moreover, the key impact of these positions in particular only became apparent after SenseNet^20^ analysis identified them to be among the strongest local conformational influencers. Indeed, in previous studies, these positions were always considered as part of a larger set of mutations with at least 4 and 6 comutations in Yi et al.,^18^ and Packer et al.,^17^ respectively. Notably, SenseNet^20^ identified M235 as the strongest contributor to local conformational changes. We decided not to explore mutagenesis for this position, as it was not in direct contact with the P1’ residue, though future studies could investigate the impact of mutations at this position. We also did not do a complete analysis of the individual contribution of each mutation; however, with all variants containing a phenylalanine at position 218, we believed this to be the most important one. This was confirmed by analyzing the effect of this amino acid within the background of three different mutants (Figure S14), which showed the strong increase in acceptance by M218F alone.

Overall, SenseNet^20^ proved to be a valuable tool for identifying positions beyond the direct interactions with the substrate. While originally developed for determining allosteric interactions, its application can be extended to identify distal sites that are connected to the active center.^28^

The unlocked promiscuity of our variant at P1’ was established by assessing catalytic efficiencies for fluorogenic peptides as well as Switchtag-Teriparatide substrates. The fluorogenic peptides were used to investigate if TEVp could tolerate previously disfavored residues at the P1’ position when this amino acid is easily accessible. In initial testing, only P1’G and P1’V were investigated as examples of tolerated and nontolerated residues, and the clone displaying the greatest improvement in P1’V cleavage was chosen for further analysis. As such, our final variant was driven by improvements for P1’V, and a different variant might have been chosen if initial testing was focused on another nontolerated P1’ residue. Still, the selected mutations displayed enhanced cleavage rates for most disfavored residues, showing that the increased tolerance they achieve goes beyond valine. Inspired by these results, the Switchtag-Teriparatide substrates with more sterically hindered P1’ accessibility were tested. Substrates of this type concurrently showed enhanced cleavage activity of TEVp-C1 versus stTEVp for most of the tested P1’ residues, underlining the universal value of the selected variant (Figure 1B). Only the cleavage rates for P1’K and P1’P did not improve for TEVp-C1 compared to stTEVp after 1 h incubation. The difficulty in cleaving P1’P was expected, as previous work failed to see cleavage for this residue even after 24 h.^1^ In that regard, it is interesting to note that after 16 h of incubation, SDS-PAGE and densitometric quantification did show elevated cleavage for P1’P in TEVp-C1. The reason why P1’K processing did not improve for TEVp-C1 after 1 h and even displayed a lower rate than stTEVp after 16 h remains speculative. While it is tempting to postulate that the loss of a negative charge to balance P1’K with D148N in TEVp-C1 is involved, this cannot be the main factor, as cleavage of the positively charged arginine at P1’ is greatly improved in TEVp-C1. While further studies could improve P1’K specificity, TEVp-C1 remains capable of cleaving P1’K, so it was deemed outside the scope of the current objective to explore this avenue.

From a mechanistic perspective, long MD simulations revealed a clear correlation between the experimentally measured cleavage rates of TEVp-C1 and stTEVp and the binding pose of fluorogenic peptides with P1’G or P1’V. Specifically, a dramatic effect of a bulky valine in the P1’ position was found for stTEVp such that it prohibits direct proton transfer in the proteolysis reaction. TEVp-C1, instead, exhibited a certain plasticity that minimizes steric effects and retains the ability for direct proton transfer after the acylation step. While analysis of the total protein:substrate interactions per substrate residue (Supplementary Figure 8) did not yield well-defined conclusions, the effect of the D148N mutation in particular was striking. The change from D148 making strongly unfavorable interactions with the substrate in stTEVp to N148 making strongly favorable interactions in TEVp-C1 underlines its value in accepting P1’V. The mechanistic analysis furthermore showed that the most preserved H-bond interactions in stTEVp are those established between P1 and its surroundings (i.e., the S1 site). Therefore, it seems that the architecture of the S1 site determines the place where the bond must be scissile. Indeed, the respective lack and abundance of H-bonding with Q7 of the substrate is clearly correlated with cleavage activity of stTEVp-P1’V and TEVp–C1-P1’V. Finally, it should be noted that the interaction visible between P11’ and M218 in the SenseNet analysis suggests that P11’ may be important in P1’V acceptance as well. However, while it can play a role, this interaction cannot be vital for P1’V acceptance, as no P11’ exists in the fluorogenic peptides tested in this study.

Taken together, we show that the selected mutations T22 V, T30A, D148N, and M218F in concert with previously engineered mutations T17S, N68D, I77 V, R203G, S219, and Δ238–242 unlock promiscuity for TEVp toward the P1’ position. The widely increased cleavage activity for different P1’ residues across different substrate types of fluorogenic peptides and Switchtag-Teriparatide proteins suggests a clear value for TEVp-C1 as a general platform to produce peptides and proteins with native N-termini. Moreover, it shows promise of the current approach combining SenseNet^20^ analysis and smart library design to identify and suggest mutant candidates for semirational protein engineering. Finally, the structural and mechanistic insights presented in the current study extend our understanding of the TEVp cleavage reaction, which may help in further efforts to engineer this protease and a key tool in chemical biology.

## Methods

### Initial Computational Investigation and SenseNet Analysis

#### *In Silico* Mutagenesis

A crystal structure of inactive TEVp, in its C151A variant, complexed with a TENLYFQSGT substrate (PDB^21^ ID: 1LVB^22^) was first subjected to amino acid substitutions using PyMol’s^29^ mutagenesis function without MD refinement at the P1’S. This provided a general insight into the local environment around the P1’ position and the residues that may sterically clash to hinder tolerance at this position.

#### Molecular Dynamics

For SenseNet analysis, the 1LVB model was further prepared to run MD simulations of stTEVp with a Switchtag-Teriparatide substrate containing a -GGSENLYFQSVS- sequence. To this end, chain A and C (containing one monomer and the substrate TENLYFQSGT with everything else removed) were aligned to a FASTA sequence of the stTEVp variant and the substrate upon which 100 candidate homology models were built using MODELER^30^ v9.18. The model with the best DOPE score was subsequently selected for MD simulations performed with the Amber18-AmberTools18 software suite^31^ using the Amber14SB force field^32^ and TIP3P water^33^ (solvating in an octahedral water box with minimum solute-face distance of 20 Å and added counterions to neutralize the system). A similar procedure was used to create and prepare the model with a P1’V substrate upon which both systems were equilibrated. Equilibration involved preliminary minimization, adjustment of the volume of the solvent box to a solvent density of 1.00 g/mL, and gradual heating of the system to 300 K over 1.01 ns (Langevin thermostat, collision frequency = 4 ps^–1^) followed by 0.5 ns unrestrained NPT simulation (Berendsen thermostat with isotropic position scaling, thermostat unchanged). Production MD was then run in the NPT ensemble under the same conditions. All simulations used a 1 fs time step and the SHAKE algorithm^34^ to constrain hydrogens.

#### SenseNet Analysis

The P1’S and P1’V trajectories were analyzed after 195.0 and 234.0 ns, respectively. First, to reduce bias to the starting structures, the first 100 ns of each simulation was removed. Next, the information required for SenseNet analysis was extracted by postprocessing the trajectories with CPPTRAJ^35^ using the “nativecontacts” and “hbond” (distance cutoff 3.5 Å, angle cutoff 135°) commands. The outputs from these commands were then combined into a network AIF file using AIFgen (v.1.0.9), which was imported into Cytoscape^23^ (v3.8.2) using the SenseNet^20^ plugin (skipping timelines <0.1avg, removing edges <0.1 occ.). The network shown in this paper was obtained using the network AIF file for the P1’V simulation, followed by calculating the edge correlation factor (ECF) scores using the “Correlation” function set to “Mutual information” mode in SenseNet.^20^ “Edge auto style” (changing width based on the “sen/correlation factor”) and “Node auto style” (changing color based on “sen/degree”) were then selected to visualize the network. Next, the nodes containing the ENLYFQV sequence were selected, and a new network was created using the first neighbors of the selected nodes (all edges). The final figure was then obtained by displaying the interactions of this smaller network using the “Degree” function weighted by “sen/correlation factor”.

### Smart Library Preparation

For library generation and protein expression, the TEV gene was in plasmid PSF593. A gene library was prepared using the primers shown in Supplementary Table 1. Freshly prepared plasmid PSF593 was used as a template in the following PCR reactions: (a) TEVUP and a 1:1 mixture of TEV 1d1 and TEV 1d2, (b) a 1:1 mixture of TEV1u1 and TEV1u2, along with a 3:1:1 mixture of TEV 2d1, TEV 2d2, and TEV 2d3, (c) a 3:1:1 mixture of TEV2u1, TEV2u2, and TEV2u3, combined with a 3:1 mixture of TEV 3d1 and TEV 3d2 and (d) a 3:1 mixture of TEV3u1 and TEV3u2, together with TEVDN. All PCR products were gel purified, and a subsequent PCR was conducted with a mixture of these fragments at approximately the same ratio as templates using TEVUP and TEVDN as primers. Different control experiments were performed by omitting one fragment as a template each time. The final PCR product was inserted into PSF593 by restriction cloning using *Bam*HI and *Hin*dIII. *E. coli* DH10b was transformed with the ligation mixture. Ca. 1500 colonies were obtained, pooled, and plasmid DNA extracted to be used for screening. To verify the quality of the library, a total of 20 variants were picked and sequenced. All variants showed only the allowed amino acids at the relevant positions, with all of them being present at least once.

### High-Throughput Screening Protocol for TEV Protease

#### Cell Culture

The mutagenic library was transferred into the BL21 *E. coli* competent cells using pRIL as a coexpression plasmid. Individual clones were picked and inoculated in sterile 96-well plates (Greiner Bio-One, GmbH, Austria), referred to as master plates, containing 100 μL of LB media, supplemented with 34 μg/mL Chloramphenicol +30 μg/mL Kanamycin, per well. Plates were sealed with parafilm and incubated at 37 °C, 250 rpm, and 80% relative humidity in a humidity shaker (Minitron, Infors, Switzerland) overnight. In each plate, column 6 was inoculated with the parental type single mutant (SM), and one well (H1-control) was inoculated with p8ref (TEV protease without activity). After 24 h incubation, plates were replicated with cryo-replicator into new 96-deep well plates containing 300 μL of TB media supplemented with 34 μg/mL Chloramphenicol + 30 μg/mL Kanamycin and 2 mM MgCl_2_. Plates were incubated at 37 °C, 250 rpm and 80% relative humidity. When OD_600_ reached 0.6–0.8., 100 μL of TB media supplemented with 34 μg/mL *Cm* + 30 μg/mL Km + 2 mM MgCl_2_ and IPTG in a final concentration of 0.1 mM was added to each well. Plates were further incubated for 19 h at 250 rpm and 80% relative humidity at 25 °C.

#### Cell Disruption

Plates were centrifuged (Eppendorf 5810R centrifuge, Germany) for 30 min, 3500 rpm at 4 °C, and the supernatant was discarded. In each cell pellet, 150 μL of BugBuster solution was added using a multidrop dispenser (Multidrop Combi Reagent Dispenser, Thermo Scientific, USA), and plates were shaken in a plate shaker until the pellet was resuspended. Finally, plates were incubated in a humidity shaker for 20 min at 25 °C and 250 rpm and centrifuged for 40 min at 3500 rpm at 4 °C to obtain the lysate solution.

#### Screening Assay

Twenty μL aliquot of lysate was transferred from the 96-deep well plate using a robotic station (Freedom EVO Tecan, Switzerland) to a microplate already containing 80 μL of high purity water (1:5 dilution). Afterward, from the 1:5 dilution microplate, 50 μL was transferred by the robot to a new microplate containing 50 μL of assay buffer 2× (100 mM Tris-HCl, 1 mM EDTA) (1:10 dilution). Next, 25 μL of 1:10 dilution was transferred to empty opaque microplates, 25 μL of previously prepared substrate solution was added, and fluorescence (Ex/Em = 340/490) was measured immediately in a plate reader (SPECTRAMax Plus 384, Molecular Devices, USA). The substrate solution of each peptide was composed of 50 mM Tris-HCl pH8.0, 0.5 mM EDTA, 1 mM DTT, and 10 μM of the corresponding fluorogenic peptide. The values were normalized against the SM values of the corresponding plate.

#### Rescreening

To rule out the selection of false positives, plasmids were extracted from winner clones, and BL21 *E. coli* was transformed again as described before. Eight individual colonies of each selected clone were inoculated in sterile 96-well plates. Again, for reliable comparison, column 6 was inoculated with the parental type stTEVp. Culture growing and screening assays were done as described before.

### Selection of Winner Mutants and Further Screening for P1’ Tolerance

#### Expression of TEVp Variants

Precultures of *E. coli* BL21 cells transformed with plasmids containing the genes of the SM and clone 1 TEVp variant were grown from 5 μL cryoculture in 25 mL 2YT medium containing 100 μg/mL ampicillin for selection in 250 mL shaking flasks. The cultures were cultivated at 37 °C and 180 rpm overnight.

On the next day, 1 L of selective 2YT medium in 5 L shaking flasks was inoculated with precultures to an initial OD_600_ of 0.1 in 5 L shaking flasks. The cell cultures were grown at 30 °C and 180 rpm until OD_600_ of 0.4–0.6 were reached, and protein expression was induced with 0.1 M IPTG. The cells were cultivated at 25 °C and 180 rpm overnight and harvested by centrifugation at 16,000*g* and 4 °C for 30 min (Avanti JXN-26, Beckman Coulter GmbH).

The cell pellets were resuspended in 50 mM Tris/HCl pH 7.3, 250 mM NaCl, 0.5 mM EDTA, 5 mM DTT, 1 mM PMSF, and 5% (w/v) glycerol and disrupted by high-pressure homogenization (LM-20, Microfluidics) for three cycles at 1200 bar. Crude cell lysates containing soluble TEV protease were obtained after centrifugation at 16,000*g* and 4 °C for 20 min.

#### Purification of TEVp Variants

Crude cell lysates were supplemented with 1 mM imidazole, and both TEV protease variants were purified by immobilized metal affinity chromatography (IMAC) using the KTA pure chromatography system. The cell lysates supplemented with 1 mM imidazole were loaded onto a cOmplete His-Tag purification column (Roche) pre-equilibrated in 50 mM Tris/HCl pH 7.3, 250 mM NaCl, 0.5 EDTA, 5 mM DTT, 5% (w/v) glycerol, and 1 mM imidazole. Bound His_6_-tagged TEV proteases were eluted in a step gradient with 30% buffer B, which corresponds to a buffer containing 300 mM imidazole. The elution signals were monitored by UV absorbance at 280 nm. Elution fractions containing TEV protease were pooled, and the buffer was exchanged for a buffer with low salt content (50 mM Tris/HCl pH 7.3, 0.5 mM EDTA, 5 mM DTT, 5% (w/v) glycerol) by gel filtration (HiPrep 26/10 Sephadex G-25, Cytiva). Remaining impurities were separated from the TEV protease by cation exchange chromatography (CEX) employing the Capto SP ImpRes column (Cytiva) pre-equilibrated in 20 mM Tris/HCl pH 7.5, 1 mM EDTA, 5 mM DTT, 5% (w/v) glycerol. Bound TEV proteases were eluted by a step gradient with 10% buffer B, which corresponds to buffer containing 100 mM NaCl. The elution signals were monitored by UV absorbance at 280 nm.

#### TEVp Activity against Fluorogenic Peptide Substrates

Purified stTEVp and TEVp-C1 variants were tested against eight fluorogenic peptide substrates to assess their cleavage efficiency against various amino acid residues in the P1’ position. All eight synthetic peptide substrates contained the peptide sequence ENLYFQXGGK (where X is represented by residues G, K, R, E, I, V, T, and L). The peptides were labeled with an EDANS fluorophore and a Dabcyl quencher at both ends and were synthesized by Pepscan (Netherlands). TEV protease cleavage between Q and X in the peptide led to the release of the EDANS fluorophore, and the fluorescence intensity can be measured at Ex/Em = 340/490 nm.

Lyophilized fluorogenic peptides were reconstituted in DMSO to obtain 1 mM stock solutions. Each substrate mix contained 50 μM peptide in reaction buffer (50 mM Tris/HCl pH 8.0, 0.5 mM EDTA, and 80 mM DTT). Purified SM and clone 1 TEVp variants were diluted to 30 μg/mL in 100 mM Tris/HCl pH 8.0, 1 mM EDTA, and 25 μL of diluted TEVp was added to 25 μL of substrate mix in black 96-well microtiter plates. The relative fluorescence unit (RFU) of the samples was measured every 30 s at 30 °C for 2 h (FLUOstar OPTIMA, BMG Labtech). The RFU was plotted against different EDANS concentrations (Supplementary Figure 9). Two time points (*T*_1_ and *T*_2_, Δ*T*) in the linear range of the plot were chosen, and the EDANS release (*B*, in pmol) was calculated based on the respective fluorescence values (RFU_2_ and RFU_1_, ΔRFU) and EDANS standard curve (Supplementary Figure 9). The TEVp activities per milligram of enzyme were calculated for each TEVp variant and fluorogenic peptide substrate based on the following formula:

$$\mathrm{TEV}p\;\mathrm{activity}\;\mathrm{per}\;\mathrm{mg}\;\mathrm{enzyme}=\frac{B}{\Delta T\times M}\times \mathrm{DF}=\frac{\mathrm{nmol}}{\min}/\mathrm{mg}$$

where *B* is the EDANs amount calculated from the EDANS standard curve (Supplementary Figure 9), Δ*T* is the difference in time points (*T*_2_ – *T*_1_) in the linear range, *M* is the amount of enzyme in the reaction, and DF is the sample dilution factor.

#### TEVp Activity Assays with Fusion Protein Substrates

Purified SM and clone 1 TEVp variants were tested against 20 Switchtag-Teriparatide fusion protein substrates containing the TEVp recognition site (ENLYFQ), each of them harboring a different canonical amino acid residue in the P1’ position. Genes encoding Switchtag-Teriparatide fusion proteins were cloned in the parental plasmid of HlyA1 ^36^ and transformed into *E. coli* BL21 (DE3) cells. Intracellular expression of Switchtag-Teriparatide as IBs, IB extraction, and denaturation in 6 M GuHCl were performed as described in Nguyen et al.^37^ The protein concentrations of the solubilized IBs were determined by UV/vis spectroscopy at 280 nm (NanoDrop One/One C, ThermoFisher) using the calculated molecular weights and extinction coefficients (ProtParam, Expasy). Renaturation of Switchtag-Teriparatide fusion proteins was performed by rapid dilution in Tris/HCl buffer (50 mM, pH 8.0, 150 mM NaCl, 10 mM CaCl_2_, 0.5 mM EDTA) and the protein concentration of each substrate was adjusted to 1 mg/mL. Purified SM and clone 1 TEVp variants were added to refolded Switchtag-Teriparatide substrates in a molar enzyme to protein ratio of 1:25, respectively. The cleavage reactions were incubated at 30 °C for 1 and 24 h. After incubation, the cleavage reactions were immediately quenched with 1:1 (v/v) 6 M GuHCl and 80 μL of cleavage samples were analyzed by RP-HPLC analysis using the 1260 Infinity II HPLC (Agilent) system and a ZORBAX SB-C18 column (80 Å, 5 μm, 4.6 mm × 250 mm). The components were eluted by a linear gradient of acetonitrile (5–60%) in water with 0.1% (v/v) TFA at a flow rate of 1 mL/min. The elution signals were monitored by the UV absorbance at 205 nm. Chromatographic elution peaks of released Teriparatide after proteolytic cleavage were integrated by the OpenLab ChemStation data software (Agilent). For visualization of differences of both TEVp variants, the values were normalized to the highest substrate protein concentration used in cleavage reactions, and the relative cleavage efficiencies were compared to SM TEVp with ENLYFQ-S (set to 100%). Cleavage reactions comprising the ENLYFQ-S substrate were additionally analyzed by RP-HPLC-MS to confirm the identity of the released target in the elution peak. The samples were injected into an Alliance HPLC system coupled to an electron spray ionization (ESI) source and a quadrupole mass spectrometer (MS, ACQUITY QDa Detector, Waters). The components were separated as described above. Following ESI in positive ion mode and quadrupole analysis, the molecular masses of the elution signals were analyzed using the Empower 3 chromatography data software.

### *In Silico* Mechanistic Studies

#### System Setup

A crystal structure of inactive TEV protease, in its C151A variant, complexed with TENLYFQSGT substrate (PDB^21^ ID: 1LVB^22^), was used to prepare two models of the TEVp-*SM* and TEVp-*C1* variants in complex with two fluorogenic peptides, ACE-E­(EDANS)-NLYFQ-**G**GK-DABCYL (SubG) and ACE-E­(EDANS)-NLYFQ-**V**G-K­(DABCYL) (SubV) as used in the experiments. For this purpose, a mutation from Ala to Cys in position 151 was reversed, and subsequently missing N-termini amino acids (highlighted in bold) **GHHHHHHGESLFKG**-^8^PRD were added using as a template the structure of the TEV protease complexed with the product (PDB^21^ ID: 1LVM). ^1^N-termini has been then modified from **GHHHHHH**-^1^GESLFKG-^8^PRD to **GTHHHHHHGSGSGT**-^1^GESLFKG-^8^PRD according to the sequence of the TEVp used in this study. Consequently, missing amino acids at C-termini (again highlighted in bold) -NKP^221^-**EEPFQPVKEATQLMN** were added using the Discovery Studio program^38^ (v.21.1.0.20298). The improved TEVp structure model was used to generate the stTEVp variant by introducing five mutations, i.e., T17S, N68D, I77 V, R203G, and S219N. All modifications in the sequence of the initial crystal structure are highlighted in Supplementary Figure 10.

The p*K*_a_ shift of all titratable residues was determined next with PropKa software^39,40^ (v.3.1). As shown in Supplementary Figure 11, all residues were found in their natural protonated state at pH 8.0. After geometrical inspection, histidine residues in positions −12, −11, −10, −9, −8, −7, 20, 61, 75, 142, and 167 were protonated in ε-, while His28 and His214 in δ-position. Disulfide bridges were not detected in the structure. Catalytic His46 was found with a slightly elevated value of p*K*_a_ (7.58), but considering that experiments were conducted at pH 8.0 and that the p*K*_a_ of Cys151 is 14.12 the His41 was deprotonated at the Nε position. Therefore, it was assumed that in the case of this cysteine protease, the active site adapts its neutral form. The protonation position was additionally verified by visual inspection of the orientation of the Cys151-His46-Asp81 triad in the active site of the crystal structure of the wild-type TEV protease.

Missing hydrogen atoms were added to the stTEVp and substrate model using the tLEAP^41^ module of the AmberTools^31^ package. Insertion of residues at both ends of the protein resulted in extended peptide chains that required optimizing their position to adapt to the rest of the protein structure. Therefore, pre-equilibration involving minimization and 500 ps of NVT molecular dynamic (MD) simulations were done in the gas phase at the molecular mechanics (MM) level using the AMBER ff03.r1 force field.^42,43^ To keep the crystal structure kernel unchanged, only residues belonging to the added termini parts were allowed to move during the simulations. The initial structure and one obtained after simulations are shown in Supplementary Figure 12. The presence of the C-end chain is especially critical because these originally missing residues seem to be involved in the formation of the binding pocket of the substrate.

The preoptimized model of stTEVp was used to build the TEVp-C1 model that was prepared by the insertion of four additional mutations, T22 V, T30A, D148N, and M218F, as predicted by SenseNet^20^ analysis. The structure of the fluorogenic peptide was prepared based on the positions of the atoms of the original substrate present in the crystal structure. As indicated in Supplementary Figure 13, both fluorophore (EDANS) and quencher (DABCYL) groups were attached to the side chains of Glu and Lys residues, respectively. Missing AMBER force field parameters for these atypical residues were generated employing Generalized Amber Force Field (GAFF),^44^ and the atomic charges were computed using the AM1 method with bond charge corrections (AM1-BCC).^45^ Parameters and atomic charges were generated using the Antechamber software^46^ and are provided in Supplementary Tables 2–3.

In the case of the stTEVp model, the overall charge of the stTEVp-P1’G and stTEVp-P1’V complexes was 0; therefore, counterions were not added to the system. In the TEVp-C1 model, the mutation of D148N caused the appearance of a positive charge that has been neutralized by the addition of one chloride (Cl^–^) counterion that was placed in the most electrostatically favorable position. Subsequently, the system was soaked within an orthorhombic box of TIP3P^33^ water molecules, with an average size of 79 × 79 × 74 Å^3^. To describe the protein and water molecules, the AMBER ff03.r1 ^42,43^ and TIP3P^33^ force fields, respectively, were employed, and the NAMD^47^ software was used as the MD engine. A cutoff for nonbonding interactions was set between 14.5 and 16 Å using a smooth switching function. The temperature during the simulations was controlled using the Langevin thermostat,^48^ and the pressure with the Nosé-Hoover Langevin piston^49^ pressure control. In all simulations, periodic boundary conditions (PBC) were applied.

#### MD Simulations

The equilibration protocol for MD simulations of four prepared models (stTEVp-P1’G, stTEVp-P1’V, TEVp–C1-P1’G, and TEVp–C1-P1’V) involved a preliminary minimization and gradual heating of the system to 303.15 K with 0.001 K temperature increments, followed by 100 ps of nonbiased NPT equilibration. The 6 μs of no-restricted unbiased NPT MD simulations with the SHAKE algorithm^34^ used to restrain all hydrogen bonds with a 2 fs time step were done. Due to the possible bias of the starting point generated by introduced mutations and missing protein fragments, the first 2 μs of MD simulations were dedicated to system equilibration, and therefore, the results from the last 4 μs of simulations were considered during the analysis of the results.

Nonbonding interaction energies were computed between the substrate and the individual residues of protein (Figure 6B) as well as the interaction energies between the full protein and the substrate, decomposed by residues of the substrate (Supplementary Figure 8). In both cases, the energies are computed as the sum of van der Waals plus electrostatic.

## Supplementary Material



## Notes

Additional experimental details and results on the structure of the binding site, validation of the screening assay, purification of the protein variants, activity analysis by different methods, various computational analyses including parameters for MD simulations, and information on DNA-primers used for library preparation (PDF)

## ○

Institute of Biochemistry, Heinrich-Heine-Universität, Universitätsstraße 1, 40225 Düsseldorf, Germany

## Abbreviations

TEVp: tobacco etch virus protease
MD: molecular dynamics
