# Supplementary material for: Engineering the Tobacco Etch Virus Protease toward a Platform for Traceless Cleavage Using Distal Site Prediction and Smart Library Design
Source: ACS Synth Biol. 2025 Aug 23;14(9):3721–33. doi: 10.1021/acssynbio.5c00423 (PMC12455634; doi:10.1021/acssynbio.5c00423)
Supplement: Supplementary file 1 [file sb5c00423_si_001.pdf]

Supporting information

Engineering the Tobacco Etch Virus protease towards a platform for traceless cleavage using distal site prediction and smart library design

Martijn P. Bemelmans[a]*, Bach-Ngan Wetzel*[b]*, Florian G. Neusius*[b]*, Florian Tieves*[b,c]*, Christian Schwarz*[b]*, Ivan Mateljak*[d]*, Katarzyna Świderek*[e]*, Vicent Moliner*[e]*, Miguel Alcalde*[f] *and Volker Sieber*[a,g,h*]

a) SynBiofoundry@TUM, Technical University of Munich, Schulgasse 16, 94315 Straubing, Germany

b) NUMAFERM GmbH, Merowingerplatz 1a, 40225, Düsseldorf, Germany

c) Current address: Institute of Biochemistry, Heinrich-Heine-Universität, Universitätsstraße 1, 40225 Düsseldorf, Germany

d) Evoenzyme S.L., Parque Cientifico de Madrid, 28049, Madrid, Spain

e) Biocomp group, Institute of Advanced Materials (INAM), Universitat Jaume I, 12071 Castello, Spain

f) Institute of Catalysis, CSIC, 28049, Madrid, Spain

g) Chair of Chemistry of Biogenic Resources, Campus Straubing for Biotechnology and Sustainability, Technical University of Munich, Schulgasse 16, 94315 Straubing, Germany

h) Catalysis Research Center, Technical University of Munich, Ernst-Otto-Fischer-Straße 1, 85748 Garching, Germany

*Corresponding author. Email: sieber@tum.de

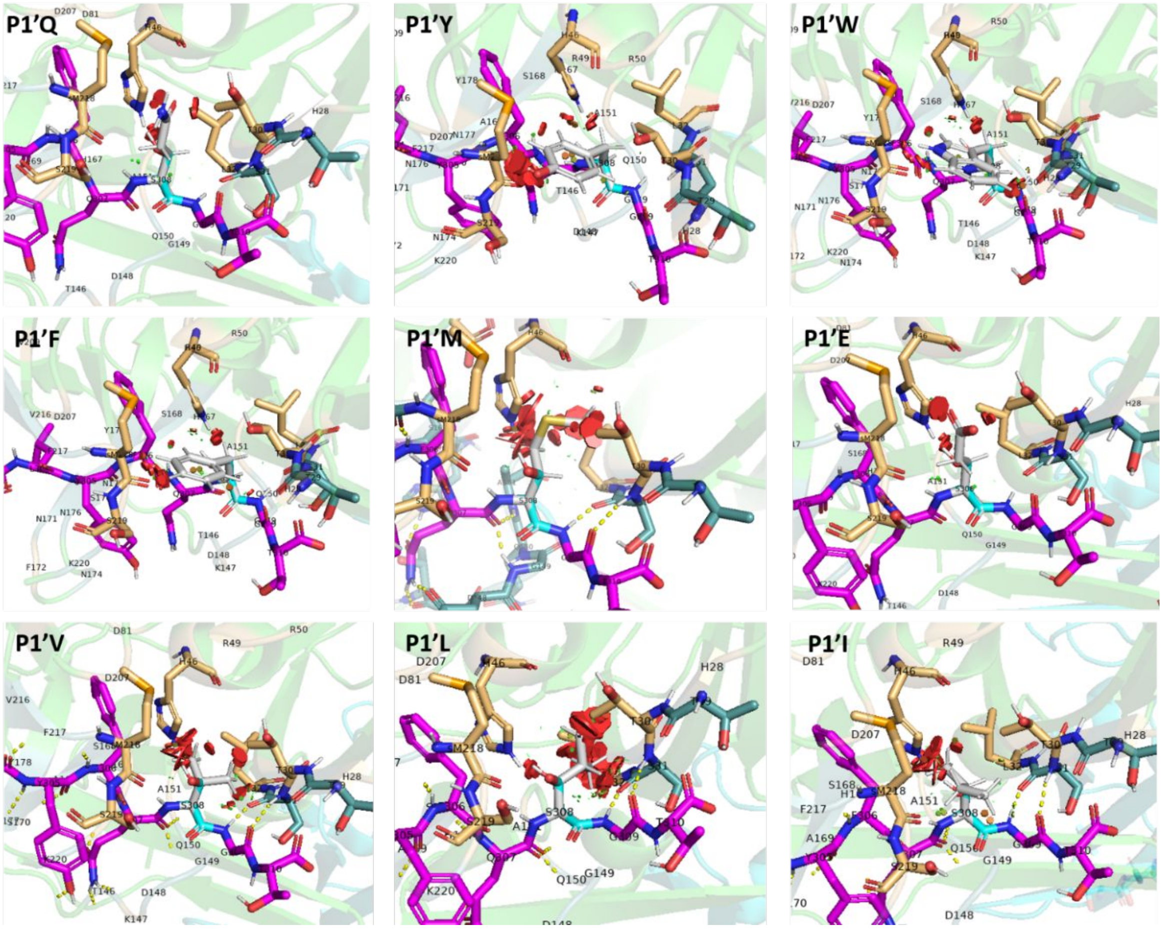

Fig. S1. Effect of *in silico* mutagenesis of substrate P1’S in the stTEVp binding site.

Different images show the steric clashes (red) predicted to arise due to mutagenesis of P1’S (cyan) to other residues (white). Based on PyMol’s mutagenesis function without MD refinement, this analysis was only used to obtain a general insight o the local environment around the P1’position. T30, L32, H46, as well as surrounding structural elements ^{28}HTTSLYGIG^{36} and ^{217}FMSKP^{221} display most clashes upon mutation.

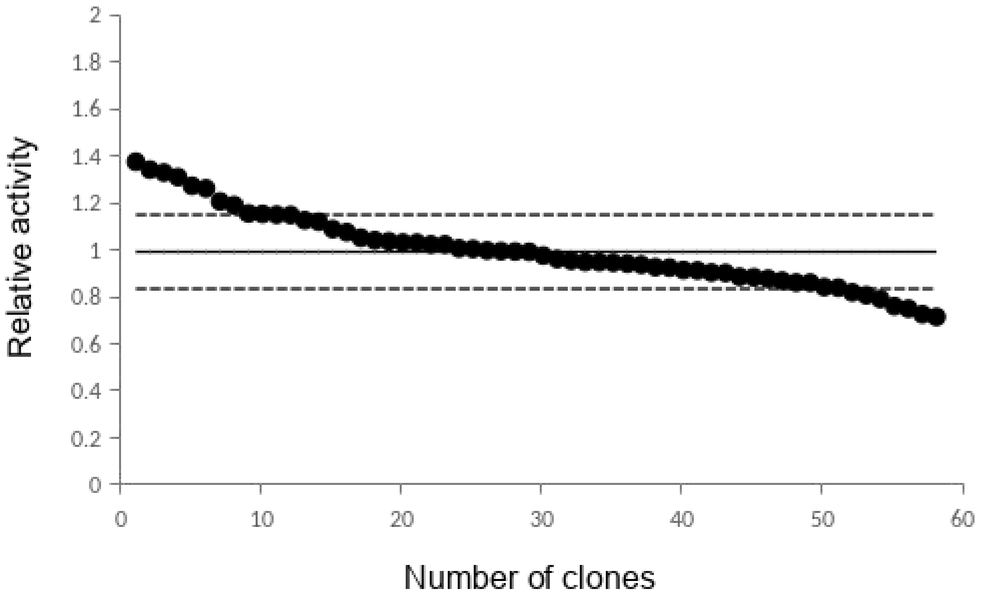

Fig. S2. Validation of screening assay.

The figure displays stTEVp assayed with ENLYFQ-G for coefficient of variation (CV). An acceptable CV of 15% was achieved.

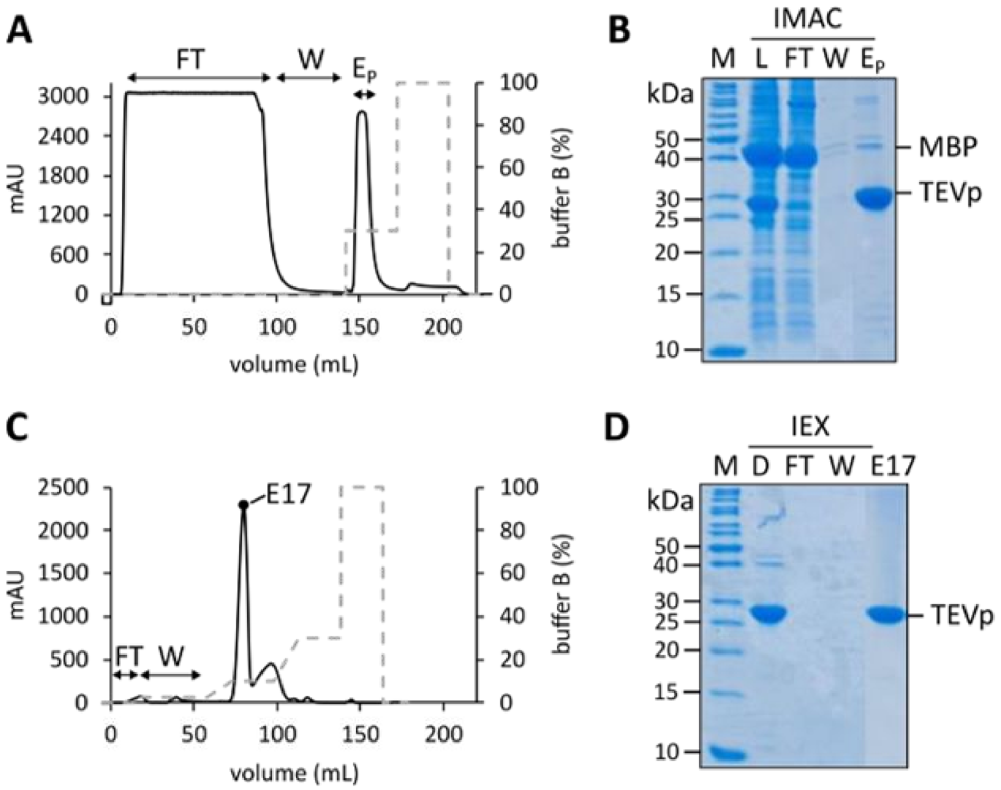

Fig. S3. Purification of parental stTEVp by IMAC and IEX

Shown are the (**A**) IMAC and (**C**) IEX chromatograms monitored at 280 nm and the coomassie-stained SDS-PAGE gels after (**B**) IMAC and (**D**) IEX purification. The gels (**C-D**) display cell lysate after cell disruption or IMAC load (L), flow through (FT), wash (W), pooled IMAC elution fractions (E_P), and molecular marker (M). The IMAC E_P fractions were desalted (D) and purified by IEX chromatography. The IEX elution fraction 17 (E17) contains the purified stTEVp variant.

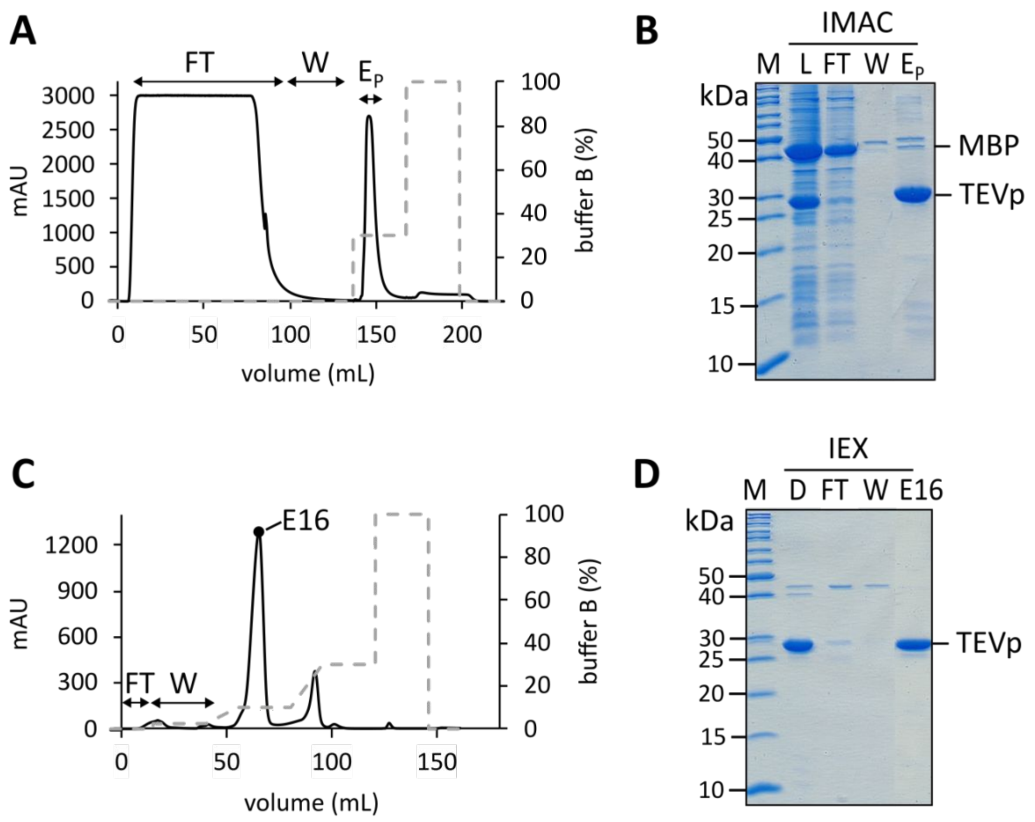

Fig. S4. Purification of the TEVp-C1 variant by IMAC and IEX

Shown are the (**A**) IMAC and (**C**) IEX chromatograms monitored at 280 nm and the coomassie-stained SDS-PAGE gels after (**B**) IMAC and (**D**) IEX purification. . The gels (**C-D**) display cell lysate after cell disruption or IMAC load (L), flow through (FT), wash (W), pooled IMAC elution fractions (E_P), and molecular marker (M). The IMAC E_P fractions were desalted (D) and purified by IEX chromatography. The IEX elution fraction 16 (E16) contains the purified TEVp-C1 variant.

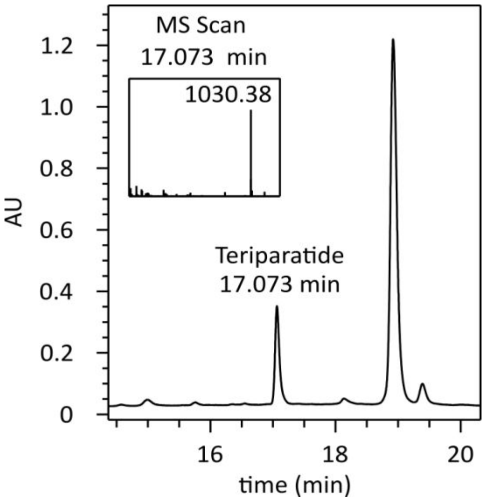

Fig. S5. RP-HPLC-MS analysis of the Teriparatide-Switchtag (ENLYFQ-S) cleavage reaction

Analytical-scale RP-HPLC-MS analysis of Switchtag-Teriparatide with the sequence ENLYFQ-S after stTEVp cleavage and incubation at 30°C for 3 h. Chromatograms show the UV absorption at 205 nm. Mass analysis of the elution signals were performed by ESI quadrupole mass spectrometry (ACQUITY QDa Detector, Waters) and confirmed the identity of Teriparatide in the elution peak at 17.073 min.

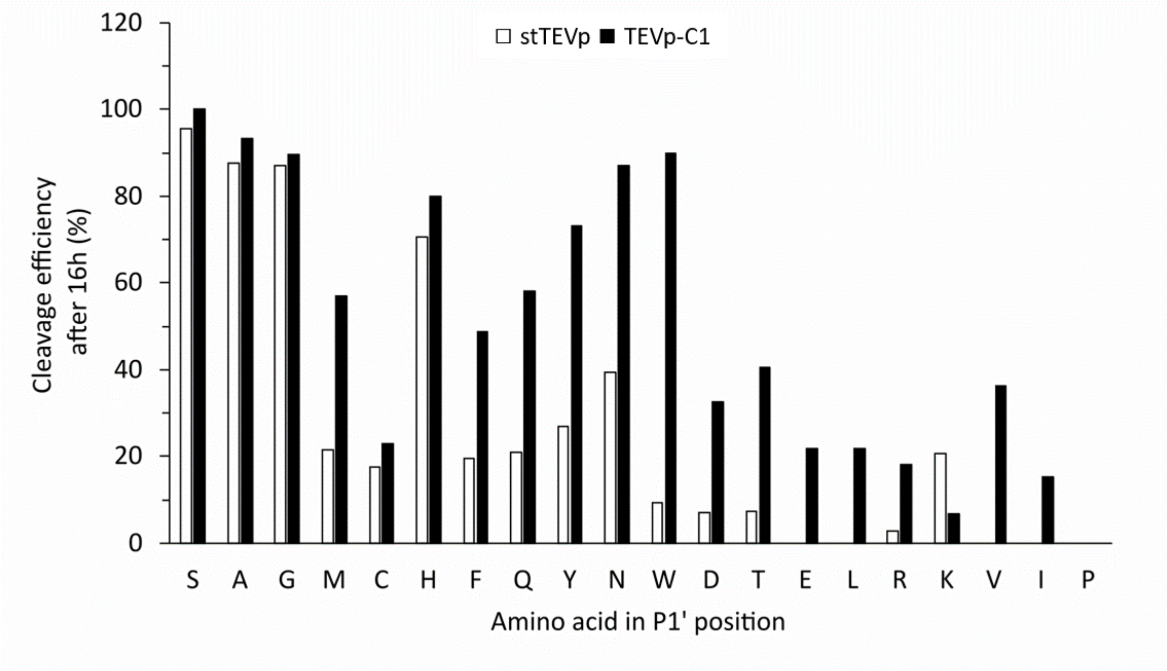

Fig. S6. TEVp activity against Switchtag-Teriparatide substrates after 16h incubation. Displaying the release of Teriparatide from the Switchtag backbone after proteolytic cleavage with stTEVp and TEVp-C1, quantified by chromatographic elution peak integration (OpenLab ChemStation data software, Agilent) after RP-HPLC. Presented as relative cleavage efficiencies (in %) compared to TEVp-C1 with ENLYFQ-S (set to 100%). Measurements were performed once.

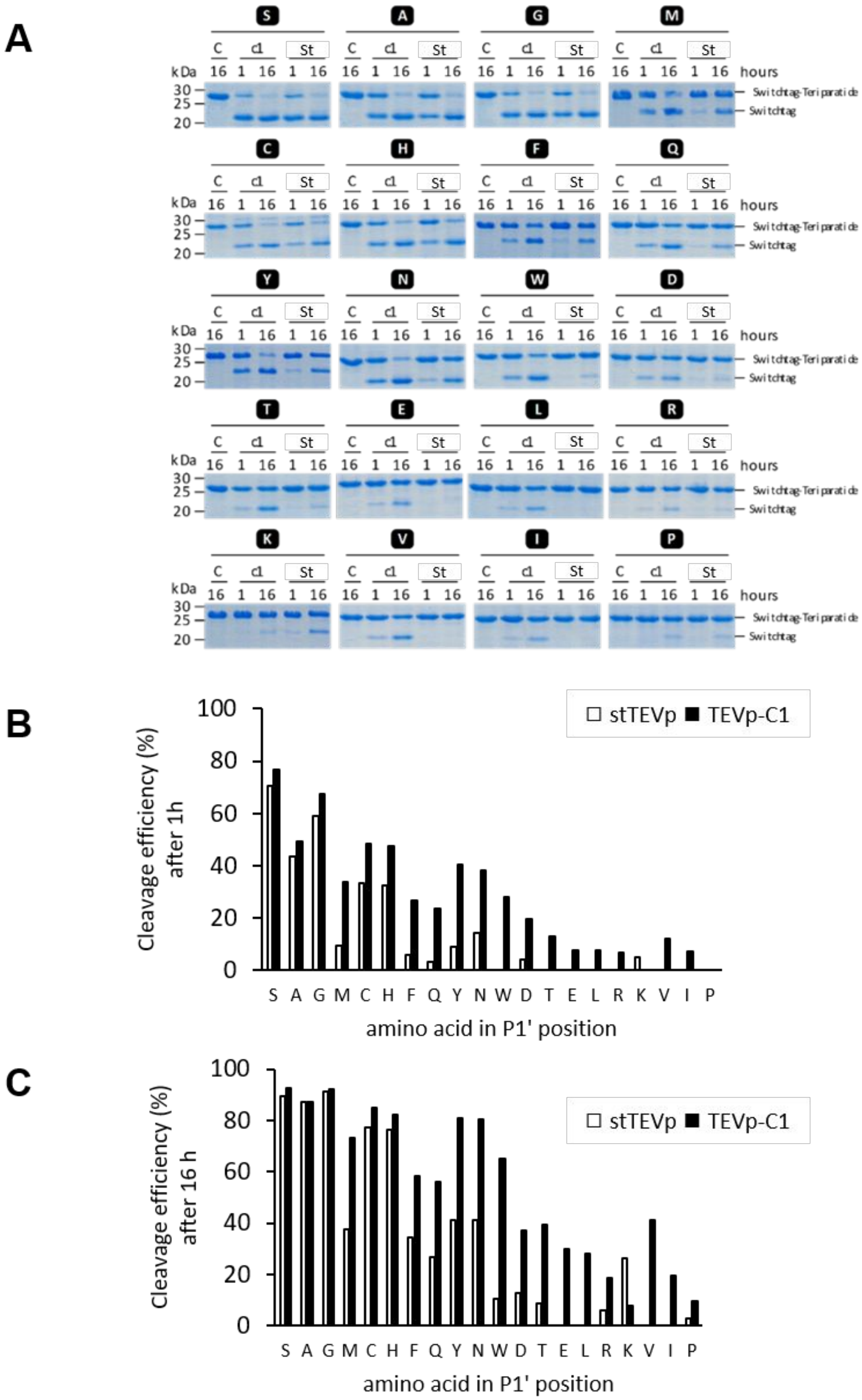

Fig. S7. SDS-PAGE analysis of TEVp activity against Switchtag-Teriparatide substrates after 1 and 16 h incubation.

A) Displays the coomassie Brilliant Blue-stained SDS-PAGE gel analysis of cleavage reactions of Switchtag-Teriparatide substrates cleaved with stTEVp (St) and TEVp-C1 (c1) variants after 1 and 16 hours incubation. Release of Switchtags after proteolytic cleavage are indicated. Switchtag-Teriparatide substrates in the absence of TEVp were incubated at 30°C for 16 h and served as control (C). **B-C)** show the cleavage efficiencies after 1h and 16h incubation, respectively, as obtained by densitometric quantification of the protein bands in **A**.

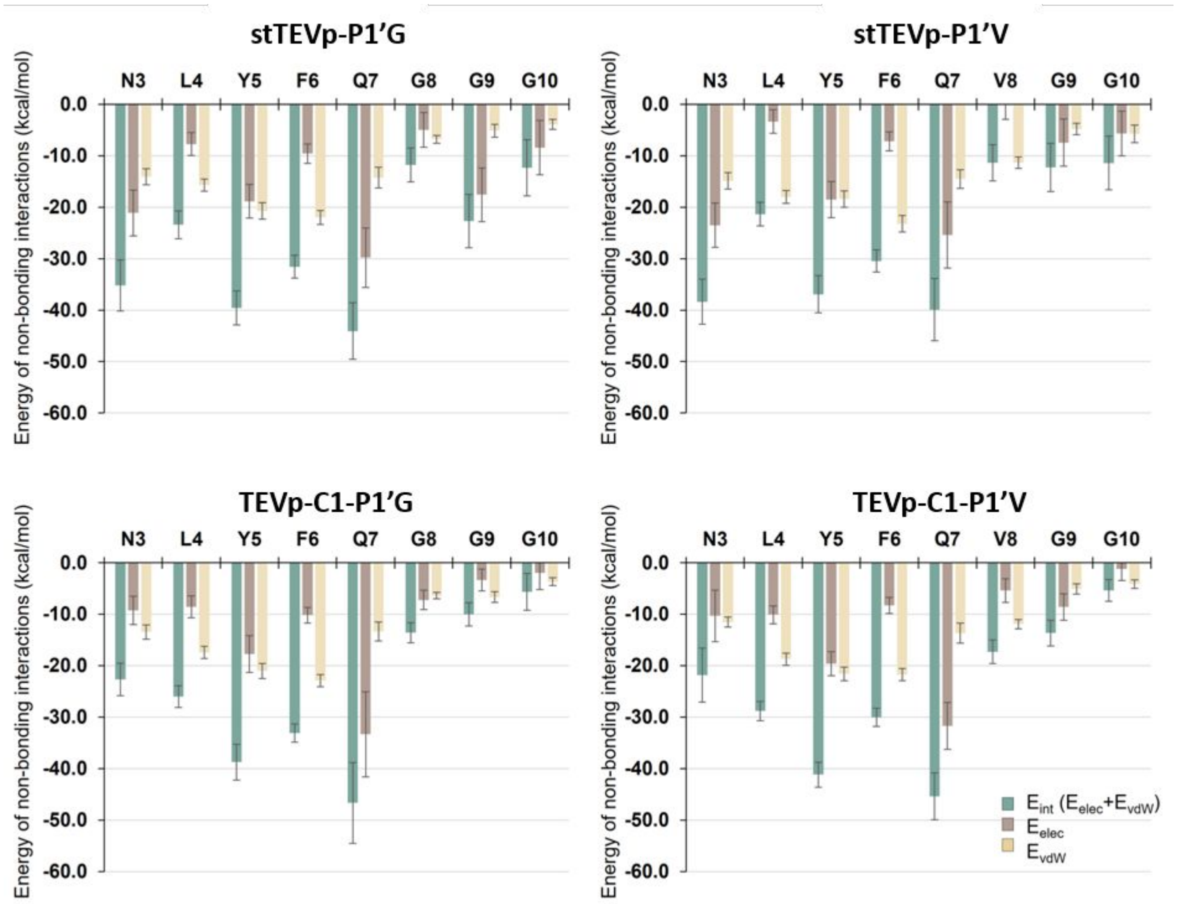

Fig. S8. Analysis of the protein:substrate interactions per substrate residue.

Non-bonded interaction energies (Van der Waals plus electrostatic) were computed between the full protein and the substrate and decomposed by residues of the substrate. Averaged values of energies are in kcal/mol and where computed from n = 3000 configurations.

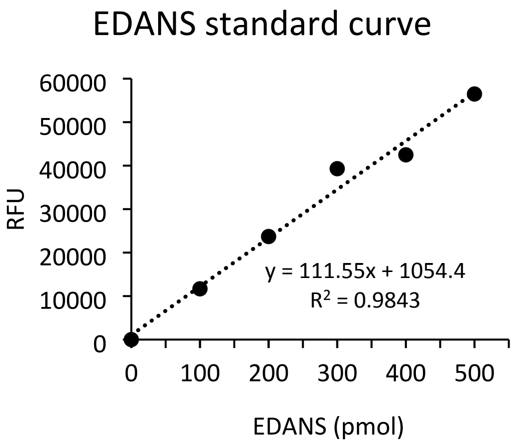

Fig. S9. EDANS standard curve.
The average of duplicates prepared for each standard measurement is shown.

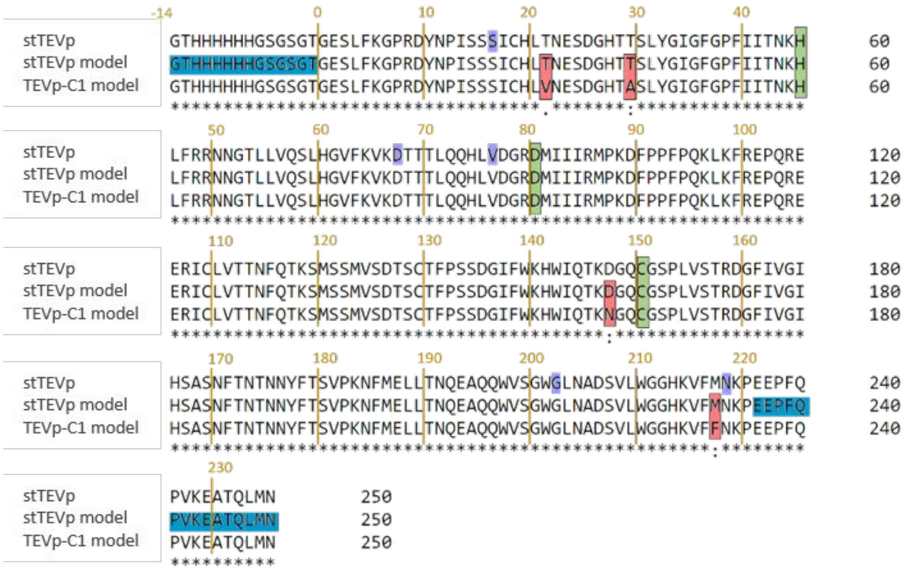

Fig. S10. Sequence alignment of the TEVp variants used in experiments and computational analysis.

Modifications introduced to the original crystalized TEVp structure at both polypeptide ends are highlighted in blue. Mutations distinguishing the stTEVp variant from the wild type are shown in purple. The differences in the sequence between the stTEVp and TEVp-C1 models are indicated in pink. The residues of the catalytic triad are highlighted in green.

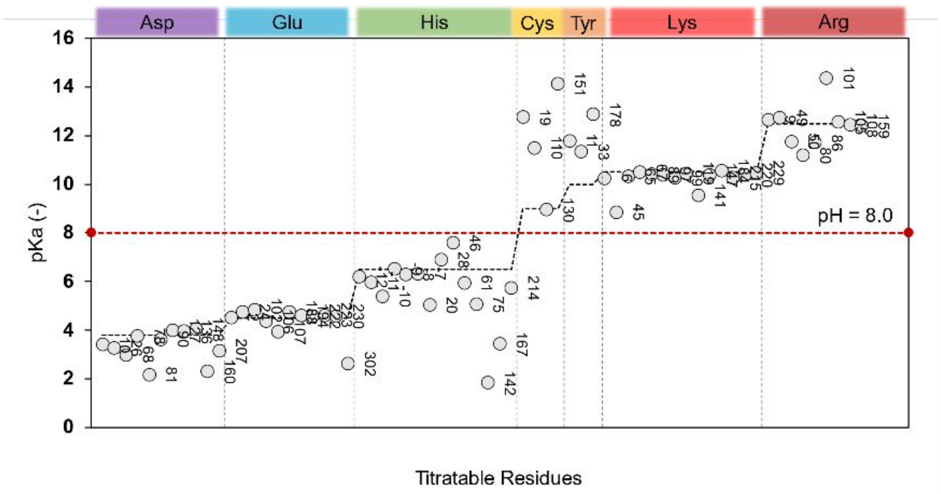

Fig. S11. Determined pKa values for titratable residues present in the stTEVp variant. Values are presented as calculated with the PropKa software.

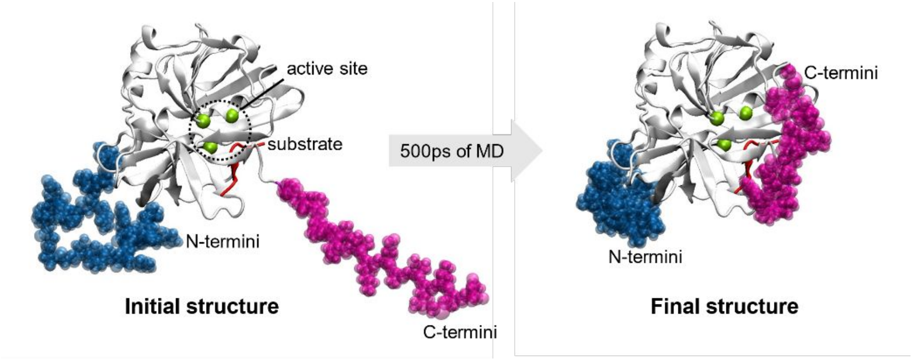

Fig. S12. Structure of the stTEVp-P1'G complex before and after initial equilibration MD simulations.

The initial and final structures are shown respectively in the left and right panel. Added N- and C-termini fragments are highlighted in blue and in magenta, while the substrate backbone is shown in red. The positions of Cα atoms of residues of the catalytic triad are indicated by green spheres.

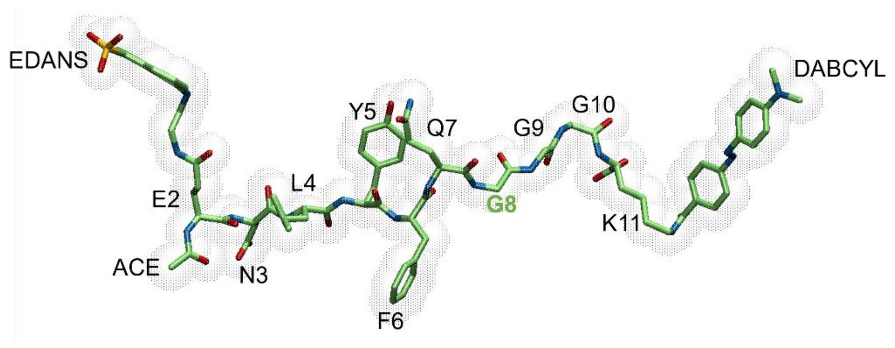

Fig. S13. Structure of the fluorogenic peptide substrate.
Carrying a P1'G (G8), with full sequence ACE-E(EDANS)-NLYFQ-**G**G-K(DABCYL).

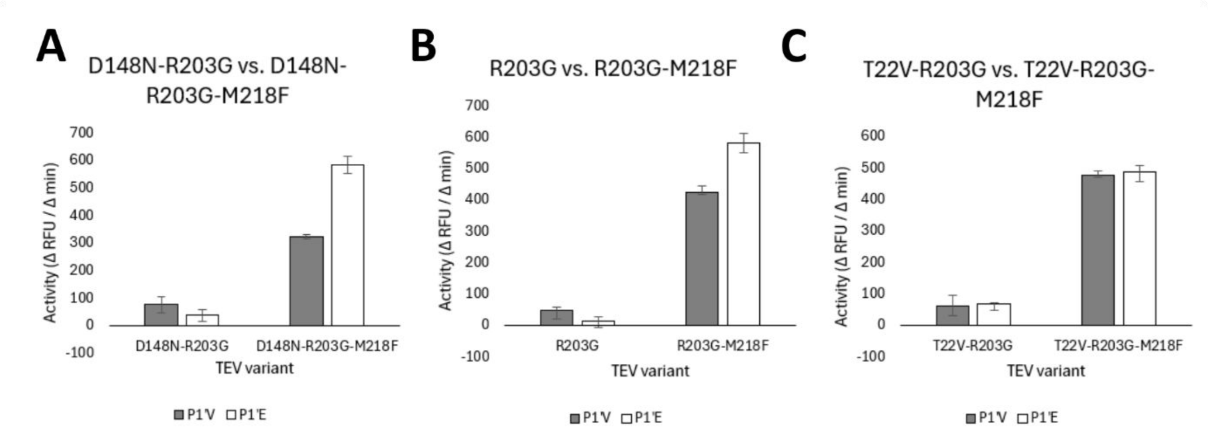

Fig. S14: Analysis on the effect of the M218F mutation on the activity of different TEV protease mutants
(A) Comparison of the activity of TEV protease variants D148N-R203G and D148N-R203G-M218F; **(B)** R203G and R203G-M218F; and **(C)** T22V-R203G and T22V-R203G-M218F. *E. coli* cells expressing the different TEV variants were cultivated in 25 cultures using 250 mL flasks, harvested by centrifugation, and lysed with BugBuster Master Mix. Following centrifugation to remove cell debris, the cell lysates containing soluble TEV proteases were diluted 5-fold in assay buffer (100 mM Tris-HCl, pH 8.0, 1 mM EDTA) and incubated with fluorogenic peptides containing either E or V at the P1' position. The activity (ΔRFU/Δmin) of each TEV variant was measured at 485 nm with excitation at 340 nm using a FLUOstar OPTIMA microtiter plate reader (BMG LABTECH).

Table S1. Primers used for gene library preparation.

No	Name	Sequence (5´→ 3´)	Type of mutation
1	TEVUP	GCAATAACAACTGCCTCG	
2	TEV1d1	GTGTGCCCATCAGATTCATT*GKA*CAAATGACAAATGGAGC	GKA reverse TMC, encoding Ser and Tyr at position 22
3	TEV1d2	GTGTGCCCATCAGATTCATT*GAY*CAAATGACAAATGGAGC	GAY reverse RTC, encoding Val and Ile at position 22
4	TEV1u1	AATGAATCTGATGGGCACACA*GMT*TCGTTGTATGGTATTGG	GMT, encoding Asp and Ala at position 30
5	TEV1u2	AATGAATCTGATGGGCACACA*AKC*TCGTTGTATGGTATTGG	AKC, encoding Ser and Ile at position 30
6	TEV2u1	CATTGGATTCAAACCAAG*DAC*GGGCAGTGTGGCAG	DAC, encoding Asp, Asn and Tyr at position 148
7	TEV2u2	CATTGGATTCAAACCAAG*CGT*GGGCAGTGTGGCAG	CGT, encoding Arg at position 148
8	TEV2u3	CATTGGATTCAAACCAAG*ATC*GGGCAGTGTGGCAG	ATC, encoding Ile at position 148
9	TEV2d1	CTGCCACACTGCCC*GTH*CTTGGTTTGAATCCAATG	GTH, reverse DAC, encoding Asp, Asn and Tyr at position 148
10	TEV2d2	CTGCCACACTGCCC*ACG*CTTGGTTTGAATCCAATG	ACG, reverse CGT, encoding Arg at position 148
11	TEV2d3	CTGCCACACTGCCC*GAT*CTTGGTTTGAATCCAATG	GAT, reverse ATC, encoding Ile at position 148
12	TEV3u1	GGGCCATAAAGTTTTC*HTC*AACAAACCTGAAGAGCC	HTC, encoding Leu, Ile and Phe at position 218
13	TEV3u2	GGGCCATAAAGTTTTC*AAA*AACAAACCTGAAGAGCC	AAA, encoding Lys at position 218
14	TEV3d1	GGCTCTTCAGGTTTGTT*GAD*GAAAACTTTATGGCCC	GAD, reverse HTC, encoding Leu, Ile and Phe at position 218
15	TEV3d2	GGCTCTTCAGGTTTGTT*TTT*GAAAACTTTATGGCCC	TTT, reverse AAA, encoding Lys at position 218
16	TEVDN	CTGAACGGTCTGGTTATAGG	

Table S2. Parameters and atomic charges for quencher DABCYL covalently attached to Lysine.

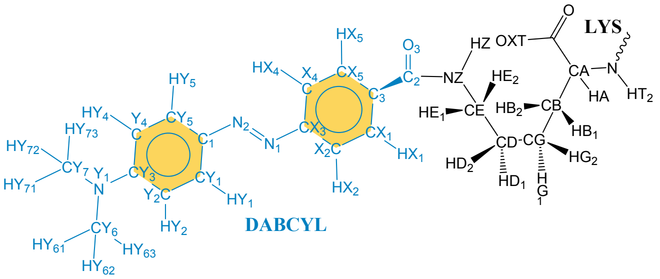

Atom name	Atom type	Charge	Atom name	Atom type	Charge
N	n3	-0.864529	HY62	h1	0.037822
HT2	hn	0.341946	HY63	h1	0.037822
CA	c3	0.028341	CY7	c3	0.190245
CB	c3	-0.055645	HY71	h1	0.037822
CG	c3	-0.084727	HY72	h1	0.037822
CD	c3	-0.090544	HY73	h1	0.037822
CE	c3	0.073171	CY4	ca	-0.193399
NZ	n	-0.512727	CY5	ca	-0.062528
C2	c	0.666476	HY5	ha	0.152010
O3	o	-0.613740	HY4	ha	0.136036
C3	ca	-0.079105	HY2	ha	0.136036
CX5	ca	-0.111968	HY1	ha	0.152010
CX4	ca	-0.098396	HX4	ha	0.145827
CX3	ca	0.084920	HX5	ha	0.153556
CX2	ca	-0.098396	HZ	hn	0.397288
CX1	ca	-0.111968	HE1	h1	0.071831
HX1	ha	0.153556	HE2	h1	0.071831
HX2	ha	0.145827	HD1	hc	0.026486
N1	ne	-0.207844	HD2	hc	0.026486
N2	nf	-0.204936	HG1	hc	0.051735
C1	ca	0.060186	HG2	hc	0.051735
CY1	ca	-0.062528	HB1	hc	0.029062
CY2	ca	-0.193399	HB2	hc	0.029062
CY3	ca	0.172725	HA	h1	0.050189
NY1	nh	-0.634001	C	c	0.905467
CY6	c3	0.190245	O	o	-0.820421
HY61	h1	0.037822	OXT	o	-0.820421

MASS			DIHEDRAL				
n3	14.010	0.530	c3-c3-c3-n3	9	1.400	0.000	3.000
hn	1.008	0.161	hc-c3-c3-n3	9	1.400	0.000	3.000
c3	12.010	0.878	o -c -c3-n3	6	0.000	180.000	2.000
n	14.010	0.530	c3-c3-n3-hn	6	1.800	0.000	3.000
c	12.010	0.616	h1-c3-n3-hn	6	1.800	0.000	3.000
o	16.000	0.434	c -c3-n3-hn	6	1.800	0.000	3.000
ca	12.010	0.360	c3-c3-c3-c3	1	0.180	0.000	-3.000
ha	1.008	0.135	c3-c3-c3-c3	1	0.250	180.000	-2.000
ne	14.010	0.530	c3-c3-c3-c3	1	0.200	180.000	1.000
nf	14.010	0.530	c3-c3-c3-hc	1	0.160	0.000	3.000

nh	14.010	0.530	o -c -c3-c3	6	0.000	180.000	2.000
h1	1.008	0.135	c3-c3-c3-n	9	1.400	0.000	3.000
hc	1.008	0.135	c3-c3-c3-h1	9	1.400	0.000	3.000
**BOND**			c3-c3-n -c	1	0.500	180.000	-4.000
hn-n3	392.40	1.019	c3-c3-n -c	1	0.150	180.000	-3.000
c3-n3	325.90	1.465	c3-c3-n -c	1	0.000	0.000	-2.000
c3-c3	300.90	1.538	c3-c3-n -c	1	0.530	0.000	1.000
c3-h1	330.60	1.097	c3-c3-n -hn	6	0.000	0.000	2.000
c -c3	313.00	1.524	o -c -n -c3	4	10.000	180.000	2.000
c3-hc	330.60	1.097	ca-c -n -c3	4	10.000	180.000	2.000
c3-n	328.70	1.462	n -c -ca-ca	4	4.000	180.000	2.000
c -n	427.60	1.379	c -ca-ca-ca	4	14.500	180.000	2.000
hn-n	403.20	1.013	c -ca-ca-ha	4	14.500	180.000	2.000
c -o	637.70	1.218	o -c -ca-ca	4	4.000	180.000	2.000
c -ca	345.90	1.491	ca-ca-ca-ca	4	14.500	180.000	2.000
ca-ca	461.10	1.398	ca-ca-ca-ha	4	14.500	180.000	2.000
ca-ha	345.80	1.086	ca-ca-ca-ne	4	14.500	180.000	2.000
ca-ne	389.30	1.408	ca-ca-ne-nf	2	0.000	180.000	3.000
ne-nf	722.40	1.263	ca-ne-nf-ca	1	3.000	180.000	-2.000
ca-nf	389.30	1.408	ca-ne-nf-ca	1	2.800	0.000	1.000
ca-nh	417.90	1.386	ha-ca-ca-ha	4	14.500	180.000	2.000
c3-nh	326.60	1.464	ha-ca-ca-ne	4	14.500	180.000	2.000
**ANGLE**			ca-ca-nf-ne	2	0.000	180.000	3.000
c3-c3-n3	66.000	111.040	ca-ca-ca-nf	4	14.500	180.000	2.000
h1-c3-n3	49.500	109.880	ha-ca-ca-nf	4	14.500	180.000	2.000
c -c3-n3	66.300	111.140	ca-ca-ca-nh	4	14.500	180.000	2.000
c3-n3-hn	47.400	109.290	ca-ca-nh-c3	4	4.200	180.000	2.000
c3-c3-c3	62.900	111.510	h1-c3-nh-ca	6	0.000	0.000	2.000
c3-c3-hc	46.300	109.800	ha-ca-ca-nh	4	14.500	180.000	2.000
c3-c -o	67.400	123.200	h1-c3-nh-c3	6	0.000	0.000	2.000
c3-c3-h1	46.400	109.560	o -c -n -hn	1	2.500	180.000	-2.000
c -c3-c3	63.300	111.040	o -c -n -hn	1	2.000	0.000	1.000
c3-c3-n	65.900	111.610	ca-c -n -hn	4	10.000	180.000	2.000
c -n -c3	63.400	120.690	h1-c3-n -c	6	0.000	0.000	2.000
c3-n -hn	45.800	117.680	h1-c3-n -hn	6	0.000	0.000	2.000
h1-c3-n	49.800	108.880	hc-c3-c3-n	9	1.400	0.000	3.000
n -c -o	74.200	123.050	h1-c3-c3-hc	9	1.400	0.000	3.000
ca-c -n	67.700	115.250	hc-c3-c3-hc	1	0.150	0.000	3.000
c -n -hn	48.300	117.550	o -c -c3-h1	1	0.800	0.000	-1.000
c -ca-ca	64.300	120.330	o -c -c3-h1	1	0.000	0.000	-2.000
ca-c -o	68.700	122.600	o -c -c3-h1	1	0.080	180.000	3.000
ca-ca-ca	66.600	120.020	c -c3-c3-c3	9	1.400	0.000	3.000
ca-ca-ha	48.200	119.880	c -c3-c3-hc	9	1.400	0.000	3.000
ca-ca-ne	67.800	120.610	**IMPROPER**				
ca-ne-nf	70.400	115.170	c -c3-n -hn		1.1	180.0	2.0
ca-ca-nh	70.400	115.170	ca-n -c -o		10.5	180.0	2.0
c3-nh-ca	67.800	120.610	c -ca-ca-ca		1.1	180.0	2.0
h1-c3-nh	68.300	120.950	ca-ca-ca-ha		1.1	180.0	2.0
c3-nh-c3	63.400	119.980	ca-ca-ca-ne		1.1	180.0	2.0
h1-c3-h1	49.600	109.790	ca-ca-ca-nf		1.1	180.0	2.0
hc-c3-hc	63.200	114.510	ca-ca-ca-nh		1.1	180.0	2.0
c -c3-h1	39.200	108.460	c3-c3-nh-ca		1.1	180.0	2.0
o -c -o	39.400	107.580	c3-o -c -o		1.1	180.0	2.0

**NONBON**			**NONBON**				
n3	1.8240	0.1700	nh	1.8240	0.1700		
hn	0.6000	0.0157	h1	1.3870	0.0157		
c3	1.9080	0.1094	hc	1.4870	0.0157		
n	1.8240	0.1700					
c	1.9080	0.0860					
o	1.6612	0.2100					
ca	1.9080	0.0860					
ha	1.4590	0.0150					
ne	1.8240	0.1700					
nf	1.8240	0.1700					

Atom types, AM1-bcc charges (in a.u.), and parameters generated based on GAFF force field.

Table S3. Parameters and atomic charges for fluorophore EDANS covalently attached to Glutamine.

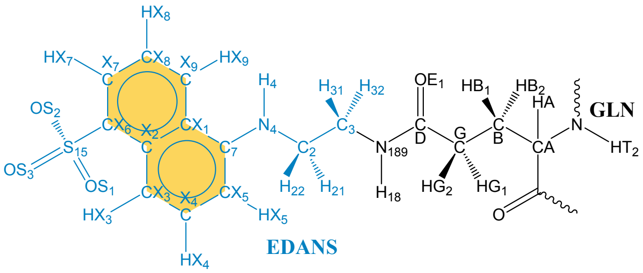

Atom name	Atom type	Charge	Atom name	Atom type	Charge
N	n3	-0.859395	OS3	o	-0.719428
HT2	hn	0.366406	OS2	o	-0.719428
CA	c3	0.053035	OS1	o	-0.719428
CB	c3	-0.073152	HX7	ha	0.163740
CG	c3	-0.161440	HX8	ha	0.114309
CD	c	0.669479	HX9	ha	0.125637
OE1	o	-0.599675	HX3	ha	0.183306
N189	n	-0.535448	HX4	ha	0.124607
C3	c3	0.043252	HX5	ha	0.112249
C2	c3	0.140878	H4	hn	0.371555
N4	nh	-0.670984	H21	h1	0.060450
C7	ca	0.075794	H22	h1	0.060450
CX5	ca	-0.179485	H31	h1	0.069203
CX4	ca	-0.139707	H32	h1	0.069203
CX3	ca	-0.089258	H189	hn	0.322845
CX2	ca	0.047371	HG1	hc	0.075897
CX1	ca	-0.047539	HG2	hc	0.075897
CX9	ca	-0.114482	HB1	hc	0.065084
CX8	ca	-0.150380	HB2	hc	0.065084
CX7	ca	-0.061122	HA	h1	0.088255
CX6	ca	-0.224599	C	c	0.542607
S15	s6	1.498476	O	o	-0.520119

MASS			DIHEDRAL				
n3	14.010	0.530	c3-c3-c3-n3	9	1.400	0.000	3.000
hn	1.008	0.161	hc-c3-c3-n3	9	1.400	0.000	3.000
c3	12.010	0.878	o -c -c3-n3	6	0.000	180.000	2.000
c	12.010	0.616	c3-c3-n3-hn	6	1.800	0.000	3.000
o	16.000	0.434	h1-c3-n3-hn	6	1.800	0.000	3.000
n	14.010	0.530	c -c3-n3-hn	6	1.800	0.000	3.000
nh	14.010	0.530	c -c3-c3-c3	9	1.400	0.000	3.000
ca	12.010	0.360	c3-c3-c3-hc	1	0.160	0.000	3.000
s6	32.060	2.900	o -c -c3-c3	6	0.000	180.000	2.000
ha	1.008	0.135	n -c -c3-c3	1	0.100	0.000	-4.000
h1	1.008	0.135	n -c -c3-c3	1	0.070	0.000	2.000
hc	1.008	0.135	c3-c -n -c3	1	0.000	0.000	-2.000
**BOND**			c3-c -n -c3	1	1.500	180.000	1.000
hn-n3	392.40	1.019	c3-c -n -hn	4	10.000	180.000	2.000
c3-n3	325.90	1.465	c3-c3-n -c	1	0.500	180.000	-4.000

c3-c3	300.90	1.538	c3-c3-n -c	1	0.150	180.000	-3.000
c3-h1	330.60	1.097	c3-c3-n -c	1	0.000	0.000	-2.000
c -c3	313.00	1.524	c3-c3-n -c	1	0.530	0.000	1.000
c3-hc	330.60	1.097	h1-c3-n -c	6	0.000	0.000	2.000
c -o	637.70	1.218	o -c -n -c3	4	10.000	180.000	2.000
c -n	427.60	1.379	o -c -n -hn	1	2.500	180.000	-2.000
c3-n	328.70	1.462	o -c -n -hn	1	2.000	0.000	1.000
hn-n	403.20	1.013	n -c3-c3-nh	9	1.400	0.000	3.000
c3-nh	326.60	1.464	h1-c3-c3-n	9	1.400	0.000	3.000
ca-nh	417.90	1.386	c3-c3-nh-ca	6	0.000	0.000	2.000
hn-nh	404.60	1.012	c3-c3-nh-hn	6	0.000	0.000	2.000
ca-ca	461.10	1.398	ca-ca-nh-c3	4	4.200	180.000	2.000
ca-ha	345.80	1.086	ca-ca-ca-nh	4	14.500	180.000	2.000
ca-s6	258.70	1.767	ha-ca-ca-nh	4	14.500	180.000	2.000
o -s6	512.70	1.453	ca-ca-ca-ca	4	14.500	180.000	2.000
**ANGLE**			ca-ca-ca-ha	4	14.500	180.000	2.000
c3-c3-n3	66.000	111.040	ca-ca-ca-s6	4	14.500	180.000	2.000
h1-c3-n3	49.500	109.880	ca-ca-s6-o	6	7.800	180.000	2.000
c -c3-n3	66.300	111.140	ha-ca-ca-s6	4	14.500	180.000	2.000
c3-n3-hn	47.400	109.290	ha-ca-ca-ha	4	14.500	180.000	2.000
c3-c3-c3	62.900	111.510	ca-ca-nh-hn	4	4.200	180.000	2.000
c3-c3-hc	46.300	109.800	h1-c3-nh-ca	6	0.000	0.000	2.000
c3-c -o	67.400	123.200	h1-c3-nh-hn	6	0.000	0.000	2.000
c3-c3-h1	46.400	109.560	h1-c3-c3-nh	9	1.400	0.000	3.000
c -c3-c3	63.300	111.040	h1-c3-c3-h1	9	1.400	0.000	3.000
c3-c -n	66.800	115.180	c3-c3-n -hn	6	0.000	0.000	2.000
c -c3-hc	46.900	108.770	h1-c3-n -hn	6	0.000	0.000	2.000
c -n -c3	63.400	120.690	o -c -c3-hc	1	0.800	0.000	-1.000
c -n -hn	48.300	117.550	o -c -c3-hc	1	0.000	0.000	-2.000
n -c -o	74.200	123.050	o -c -c3-hc	1	0.080	180.000	3.000
c3-c3-n	65.900	111.610	n -c -c3-hc	6	0.000	180.000	2.000
h1-c3-n	49.800	108.880	c -c3-c3-hc	9	1.400	0.000	3.000
c3-n -hn	45.800	117.680	hc-c3-c3-hc	1	0.150	0.000	3.000
c3-c3-nh	66.200	110.460	c3-c3-c3-h1	9	1.400	0.000	3.000
c3-nh-ca	63.400	119.980	h1-c3-c3-hc	9	1.400	0.000	3.000
c3-nh-hn	46.100	115.990	o -c -c3-h1	1	0.800	0.000	-1.000
h1-c3-nh	49.600	109.790	o -c -c3-h1	1	0.000	0.000	-2.000
ca-ca-nh	68.300	120.950	o -c -c3-h1	1	0.080	180.000	3.000
ca-nh-hn	48.400	116.070	o -c -N -H	1	2.500	180.000	-2.000
ca-ca-ca	66.600	120.020	o -c -N -H	1	2.000	0.000	1.000
ca-ca-ha	48.200	119.880	O -C -n3-hn	1	2.500	180.000	-2.000
ca-ca-s6	61.900	120.430	O -C -n3-hn	1	2.000	0.000	1.000
ca-s6-o	67.900	104.090	O -C -n3-c3	4	10.000	180.000	2.000
o -s6-o	73.600	120.050	c3-c -N -H	4	10.000	180.000	2.000
h1-c3-h1	39.200	108.460	CT-C -n3-hn	4	10.000	180.000	2.000
hc-c3-hc	39.400	107.580	n3-c3-c -N	1	1.700	180.000	-1.
c -c3-h1	47.000	108.220	n3-c3-c -N	1	2.000	180.000	2.
**NONBON**			o -c -N-CT	4	10.000	180.000	2.000
n3	1.8240	0.1700	c3-c3-c -N	1	0.100	0.0	-4.
hn	0.6000	0.0157	c3-c3-c -N	1	0.07	0.0	2.
c3	1.9080	0.1094	C -n3-c3-c	1	0.850	180.000	-2.
c	1.9080	0.0860	C -n3-c3-c	1	0.800	0.000	1.
o	1.6612	0.2100	c3-c3-n3-C	1	0.50	180.0	-4.

n	1.8240	0.1700	c3-c3-n3-C	1	0.15	180.0	-3.
nh	1.8240	0.1700	c3-c3-n3-C	1	0.00	0.0	-2.
ca	1.9080	0.0860	c3-c3-n3-C	1	0.53	0.0	1.
s6	2.0000	0.2500	C -n3-c3-h1	1	0.156	0.000	3.000
ha	1.4590	0.0150	X -C -n3-X	4	10.00	180.0	2.
h1	1.3870	0.0157	X -c -N -X	4	10.00	180.0	2.
hc	1.4870	0.0157	X -c -c3-X	6	0.00	0.0	2.
			**IMPROPER**				
			c3-n -c -o		10.5	180.0	2.0
			c -c3-n -hn		1.1	180.0	2.0
			c3-ca-nh-hn		1.1	180.0	2.0
			ca-ca-ca-nh		1.1	180.0	2.0
			ca-ca-ca-ha		1.1	180.0	2.0
			ca-ca-ca-ca		1.1	180.0	2.0
			ca-ca-ca-s6		1.1	180.0	2.0

Atom types, AM1-bcc charges (in a.u.), and parameters generated based on GAFF force field.
